# Facile General Injectable Gelatin/Metal/Tea Polyphenol Double Nanonetworks Remodel Wound Microenvironment and Accelerate Healing

**DOI:** 10.1002/advs.202305405

**Published:** 2023-12-20

**Authors:** Xingjie Zan, Dong Yang, Yi Xiao, Yaxin Zhu, Hua Chen, Shulan Ni, Shengwu Zheng, Limeng Zhu, Jianliang Shen, Xingcai Zhang

**Affiliations:** ^1^ National Engineering Research Center of Ophthalmology and Optometry Eye Hospital Wenzhou Medical University Wenzhou 325027 China; ^2^ Wenzhou Institute Wenzhou Key Laboratory of Perioperative Medicine University of Chinese Academy of Sciences Wenzhou 325001 China; ^3^ School of Engineering and Applied Sciences Harvard University Cambridge MA 02138 USA; ^4^ Department of Thoracic Surgery The First Affiliated Hospital of Wenzhou Medical University Wenzhou 325003 China; ^5^ Wenzhou Celecare Medical Instruments Co., Ltd Wenzhou 325000 China

**Keywords:** double nanonetworks, injectable hydrogel, metal organic interaction, remodel wound microenvironment, tea polyphenol

## Abstract

Treating the most widespread complication of diabetes: diabetic wounds poses a significant clinical obstacle due to the intricate nature of wound healing in individuals with diabetes. Here a novel approach is proposed using easily applicable injectable gelatin/metal/tea polyphenol double nanonetworks, which effectively remodel the wound microenvironment and accelerates the healing process. The gelatin(Gel) crosslink with metal ions (Zr^4+^) through the amino acids, imparting advantageous mechanical properties like self‐healing, injectability, and adhesion. The nanonetwork's biological functions are further enhanced by incorporating the tea polyphenol metal nanonetwork through in situ doping of the epigallocatechin gallate (EGCG) with great antibacterial, self‐healing, antioxidant, and anticancer capabilities. The in vitro and in vivo tests show that this double nanonetworks hydrogel exhibits faster cell migration and favorable anti‐inflammatory and antioxidant properties and can greatly reshape the microenvironment of diabetic wounds and accelerate the wound healing rate. In addition, this hydrogel is completely degraded after subcutaneous injection for 7 days, with nondetectable cytotoxicity in H&E staining of major mice organs and the serum level of liver function indicators. Considering the above‐mentioned merits of this hydrogel, it is believed that the injectable gelatin/metal/tea polyphenol double nanonetworks have broad biomedical potential, especially in diabetic wound repair and tissue engineering.

## Graphical abstract

Conceptual description of a multifunctional Gel/Zr/EGCG hydrogels to promote diabetic wound healing. A) The formation of Gel/Zr/EGCG hydrogels is obtained through metal coordination reactions between amino, carboxyl, hydroxyl, and imidazole groups on Gel and Zr^4+^. B) Gel/Zr/EGCG hydrogels dressings are constructed using a simple injection mix that exhibits antibacterial properties, reduces inflammation, promotes orderly wound healing, and guides tissue regeneration.

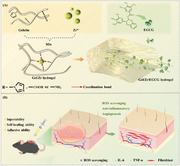

## 1 Introduction

Diabetic wounds are the most widespread complication of diabetes and have affected more than 34 million people in USA and 55 million patients worldwide and is predicted to affect more than 80 million by 2030.^[^ ^1^, ^2^, ^3^, ^4^ ^]^ These wounds are characterized by their chronicity, impaired blood vessel formation, persistent pain, bacterial infections, and heightened inflammation. More than half of diabetic wounds develop into chronic wounds with disrupted skin regeneration and high recurrence probabilities, which places a heavy economic burden on individual patients and public health systems.^[^ ^1^, ^2^, ^3^, ^4^ ^]^ Treating diabetic wounds, particularly diabetic ulcers, poses a significant clinical obstacle due to the intricate nature of wound healing in individuals with diabetes.^[^ ^1^ ^]^ We and our collaborators developed a near‐infrared‐responsive black phosphorus‐based sprayed fibrin gel capable of alleviating pain, eliminating bacteria, reducing inflammation, and promoting angiogenesis.^[^ ^1^ ^]^ Here we propose a novel approach using easily applicable injectable gelatin/metal/tea polyphenol double nanonetworks, which effectively remodel the wound microenvironment and accelerates the healing process for excessive reactive oxygen species (ROS), controlling bacterial infections, and regulating angiogenesis around diabetic chronic wounds without a drug.

Gel, an important class of bioactive materials derived from native collagen, has excellent biocompatibility, good biodegradability, and promotion of cell adhesion, proliferation, and differentiation.^[^ ^5^, ^6^ ^]^ Gel contains various chemical reactive residues, including primary amines, carboxyl groups, and hydroxyl groups, which can be further modified using various functional molecules, thereby increasing its multifunctionality in tissue retyping.^[^ ^7^ ^]^ Postmodifying Gel with dynamic crosslinked groups generates injectable Gel‐based hydrogels. However, the disadvantages of these postmodification strategies include the cytotoxicity of the chemical agents used for modifications and delicate requirements of controllable chemical reactions,^[^ ^8^, ^9^ ^]^ which largely limit the biomedical applications of Gel‐based hydrogels. Moreover, the complicated and time‐consuming purifications and modifications likely destroy the bioactive sequence of Gel.^[^ ^10^ ^]^ Therefore, a simple and convenient strategy to produce Gel‐based injectable hydrogels is highly desirable.^[^ ^11^ ^]^

**Figure 1:**
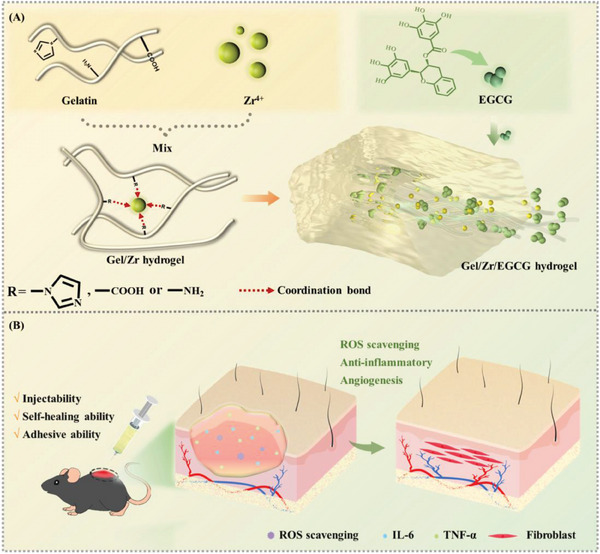
Conceptual description of a multifunctional Gel/Zr/EGCG hydrogels to promote diabetic wound healing. A) The formation of Gel/Zr/EGCG hydrogels, which was obtained through metal coordination reactions between amino, carboxyl, hydroxyl, and imidazole groups on Gel and Zr^4+^. B) Gel/Zr/EGCG hydrogels dressings were constructed using a simple injection mix that exhibited antibacterial properties, reduced inflammation, promoted orderly wound healing, and guided tissue regeneration.

Metal ions have multiple biological functions^[^ ^12^ ^]^: Ca is beneficial for bone formation,^[^ ^13^ ^]^ Cu promoted angiogenesis at low concentrations,^[^ ^14^ ^]^ and Ag and Zn have promising antibacterial properties,^[^ ^15^, ^16^ ^]^ Mg has been approved by the FDA for clinical bone repair and the treatment of stomach diseases, and Fe has also been approved by the FDA for the treatment of iron deficiency.^[^ ^17^ ^]^ Recently, metal ions were introduced into Gel‐based hydrogels via three steps: 1) conjugating the radical polymerizable group onto a coordinative monomer and Gel, 2) forming the hydrogel using a free radical initiator and UV irradiation, and 3) soaking the hydrogel into a solution of metal ions.^[^ ^18^ ^]^ However, this process produces noninjectable Gel‐based hydrogels that also require complicated post‐chemical modifications and time‐consuming purifications. Coordinative interactions between metal ions and proteins are ubiquitous and essential for the biological functions of proteins,^[^ ^19^ ^]^ including biocatalysis,^[^ ^20^ ^]^ biosignaling,^[^ ^21^ ^]^ and nutrient transport. Gel residues contain various groups, such as carboxyl, amine, and imidazole groups,^[^ ^22^ ^]^ which can form coordinative interactions with metal ions. Hence, we hypothesized that Gel‐based injectable hydrogels could be generated in a facile and easy manner by directly mixing metal ions and Gel (**Figure** **1**A). To test this hypothesis, several metal ions were investigated for their abilities to generate Gel hydrogels and those with multiple coordination modes with proteins were extensively explored. Our data demonstrated that by optimizing pH and metal ion and Gel concentrations, injectable, self‐healing, and adhesive Gel‐based hydrogels could be obtained by coordinating the residual carboxyl and amino groups in Gel with metal ions such as zirconium (Zr^4+^) or ferric (Fe^3+^) ions.

Epigallocatechin‐3‐gallate (EGCG), a green tea extract, is known for its ROS‐scavenging, anti‐inflammatory, bactericidal, antiaging, and proangiogenic effects.^[^ ^23^, ^24^ ^]^ Although the functions of EGCG have been known for years, it is low bioavailability and rapid metabolism remain a bottleneck to its prospective application.^[^ ^25^, ^26^, ^27^, ^28^ ^]^ Herein, EGCG was in situ doped into a zirconium ion (Zr^4+^)‐crosslinked Gel hydrogel (Gel/Zr), by which the adhesive, mechanical, antioxidant, angiogenic properties of Gel/Zr could be further improved (Figure 1B). Zr^4+^ has low cytotoxicity and excellent antibacterial properties and we demonstrated that EGCG‐doped Gel/Zr hydrogel (Gel/Zr/EGCG) effectively repaired diabetic chronic wounds in 14 days by remodeling the wound microenvironment.

Our hydrogel double nanonetworks are 3D network structures comprising crosslinked polymer chains and metal polyphenol nanonetwork with strong water absorption capacities, excellent biocompatibility, and metabolite transport capabilities, which can simulate the cellular microenvironment and natural extracellular matrix (ECM). Several tunable moduli and an abundance of materials capable of forming hydrogels can attract extensive attention from various disciplines.

## 2 Results

### 2.1 Preparation and Characterization of the Gel/Zr Hydrogel

**Figure 2:**
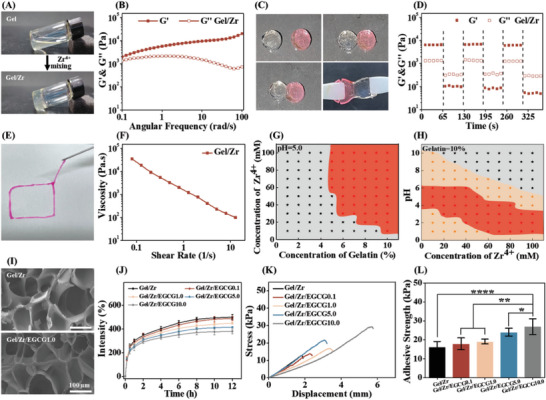
Fabrication and viscoelastic characterization of injectable Gel/Zr hydrogel. A) Images of the Gel/Zr hydrogel. B) *G*′ and *G*″ of Gel/Zr hydrogel (strain = 1%). C) Self‐healing properties of the Gel/Zr hydrogel. D) Self‐healing properties of the Gel/Zr hydrogel under alternating strain between 1% and 400% (frequency = 0.1 Hz). E) Injection of the Gel/Zr hydrogel. F) Shear thinning behaviors of the Gel/Zr hydrogel. G) Phase diagrams of the Gel and Zr^4+^ mixture at pH 5.0. H) Phase diagrams of the Gel and Zr^4+^ mixture at a 10% Gel concentration. According to the different colors of stars, they represent liquid (★), coacervate (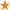), and hydrogel (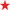). Physical characterization of the Gel/Zr/EGCG hydrogels: I) Representative cross‐sectional SEM images of the Gel/Zr and Gel/Zr/EGCG1.0 hydrogels. J) Swelling behavior of the Gel/Zr/EGCG hydrogels in PBS at 37 °C (*n* = 4). K) Tensile stress–strain curves of the Gel/Zr/EGCG hydrogels. L) The adhesive strength of Gel/Zr/EGCG hydrogels on pig skin loaded with different EGCG concentrations (*n* = 4).

By tilting the bottle to change of the liquid level (**Figure** **2**A), we observed that Gel formed a hydrogel after blending with Zr^4+^. Rheological measurements were performed to characterize the viscoelastic behavior of the Gel/Zr hydrogel, where *G*′ (elastic behavior) and *G*″ (viscous behavior) varied with the angular frequency (Figure 2B) and *G*′ showed a weak frequency dependence over the entire frequency range. The *G*′ values were higher than the corresponding *G*″ values over the entire frequency range, indicating the relatively stable crosslinked structures of Gel/Zr hydrogel. The healing properties of the Gel/Zr hydrogel were visualized using two tablet‐like hydrogels, one with Rhodamine B and one without. After 20 min, the boundaries of both hydrogels began to blur, mutual adhesion was observed, and the overall healing was demonstrated when tweezer‐assisted stretching did not introduce simultaneous breaks; the adhesion and overall healing of two kinds of hydrogels (Figure 2C) showed the excellent self‐healing capabilities of the hydrogel. Strain amplitude sweep test in Figure S1 (Supporting Information) shows that the strain at the intersection of *G*′ and *G*′′ is 105%, which indicates that the hydrogel is in the critical state of colloid and solution. When the strain exceeds the critical strain, the *G*′ of the hydrogel decreases significantly and is lower than *G*″, indicating that the polymer molecular chain in the hydrogel slips off seriously, the gel network is destroyed, and fluidity began to dominate elasticity in Gel/Zr samples, suggesting typical shear thinning features. The self‐healing properties of the hydrogel were further confirmed by alternating high and low strain scanning modes (Figure 2D). For a hydrogel strain of 400%, *G*′ immediately decreased from 4500 to 700 Pa and was lower than *G*″. When the strain returned to 1%, both *G*′ and *G*″ rapidly returned to their initial values. The damage and recovery behavior of the crosslinked structure of the hydrogel could be repeated many times, proving the rapid and efficient self‐repairing ability of the hydrogel. The Gel/Zr system could be continuously injected into a Petri dish with a syringe needle to form a “□” font that maintained the hydrogel state (Figure 2E). Additionally, the shear thinning properties of the hydrogel were investigated as a function of the shear rate (Figure 2F). Increasing angular frequencies decreased the hydrogel viscosities by 2–3 orders of magnitude, indicating the destruction of the crosslinked hydrogel structures and their shear thinning behaviors. The shear thinning properties of the hydrogel indicated its conduciveness to being injected.

Figure 2G shows the phase diagram of the mixture of Zr^4+^ and Gel with different concentrations of Gel and metal ions at pH 5.0. Increasing Gel concentrations decreased the gelatinization concentration of Zr^4+^. Therefore, a Gel concentration of 10% was selected to explore the metal ion concentrations and gelatinization phase diagrams at different pH values (Figure 2H) and (Figure S2A, Supporting Information). Overall, an acidic pH environment was more favorable for gelatinization. Compared with that of Gel/Fe, the Gel concentration window of Gel/Zr was much wider, with Gel occurring over a wider pH range (1.5–6.0 for Gel/Zr versus 3.5–5.5 for Gel/Fe). In addition, Cu^2+^ cannot combine with Gel (Figure S2B, Supporting Information) at different pH values. Simultaneously, we also tested various common metal ions directly mixed with Gel, all of which failed to coordinate to form hydrogels (Table S1, Supporting Information). Therefore, Gel/Zr was selected for subsequent experiments. The presence of metal ions was identified by energy dispersion spectroscopy (EDS). The characteristic elemental peaks of Zr in the EDS spectra demonstrated their efficient incorporation into the hydrogel (Figure S3A, Supporting Information). EDS mapping further demonstrated that Zr ions were almost uniformly distributed within the hydrogel (Figure S3B, Supporting Information).

EGCG was in situ doped into the Gel/Zr hydrogel by premixing Gel with EGCG. The Gel of Gel/Zr/EGCG1.0 hydrogel was observed in a bottle tilting experiment (Figure S4A, Supporting Information). Rheological analysis (Figure S4B, Supporting Information) of Gel/Zr hydrogel containing 0.1, 1.0, 5.0, and 10.0 mg mL^−1^ of EGCG showed that while the doping amount of EGCG did not affect the crosslinked Gel structure, the *G*′ value of hydrogels decreased increasing EGCG concentrations (Figure S4C, Supporting Information). The Gel/Zr/EGCG5.0 hydrogel was tested as an example of the Strain amplitude sweep test (Figure S4D, Supporting Information), alternating high and low strain scans (Figure S3D, Supporting Information) and shear thinning (Figure S4E, Supporting Information), and the results showed that the Gel/Zr/EGCG5.0 hydrogel maintained its healing and injectable functions. Compared with the Gel/Zr hydrogel, the shear thinning of Gel/Zr/EGCG5.0 hydrogel was easier at the same shear force, suggesting a better healing and injectable profile. Swelling is a fundamental characteristic of hydrogels, which reflects the water absorption capacity of the hydrogel. The internal structures of the hydrogels were observed by SEM and found to be a porous uniform honeycomb structure (Figure 2I); adding EGCG has little effect on the void structure of the hydrogel. This indicates the potential of Gel/Zr/EGCG hydrogels to absorb excess tissue exudates and transport water vapor. The swelling rates of the hydrogels were determined in PBS at 37 °C over 12 h, although the water uptake capacities of the hydrogels were almost saturated after 8 h. Although the swelling rate of the Gel/Zr hydrogel decreased with an increasing EGCG content, the swelling equilibrium values were all higher than 400%, indicating their ideal water absorption ability (Figure 2J). The rapid swelling of Gel/Zr/EGCG hydrogels may be attributed to its porous structure and internal hydrophilic groups.

The application of hydrogel dressings with appropriate adhesive strengths to wound sites can create a highly effective microenvironment for wound healing. To further explore the adhesion of Gel/Zr hydrogel to the skin, the adhesion of the Gel/Zr hydrogel was evaluated using a lap shear test with porcine skin as the substrate (Figure S5A, Supporting Information). The maximum adhesive strength of both pieces of porcine skin was used to determine the adhesion effect of the hydrogel (Figure 2K). The adhesion strength was significantly enhanced for higher EGCG concentrations, which was consistent with previous reports on catechol groups with strong adhesion effects on various substrates.^[^ ^29^ ^]^ The adhesion strength of the hydrogel increased from 16.2 to 26.9 kPa (Figure 2L), indicating that EGCG significantly enhanced the adhesion of the hydrogel to biological tissues. Next, the enhanced adhesion of Gel/Zr/EGCG hydrogels was verified for different substrates, including glass, plastic, and metal (Figure S5B, Supporting Information). These data indicate that Gel/Zr/EGCG hydrogels firmly adhere to wounds to form a rational porous honeycomb barrier, thus providing a suitable microenvironment to potentially accelerate diabetic wound repair.

### 2.2 Interaction Modes within the Gel/Zr/EGCG Hydrogels

Based on the rich functional groups of gel and polyphenols, the gel, polyphenol, and metal system may exhibit different noncovalent interactions between gel and polyphenols, including hydrogen bonding and ionic and hydrophobic interactions, as well as coordination bonds between the gel, polyphenols, and metals. Fourier transform infrared spectroscopy (FTIR) and X‐ray photoelectron spectroscopy (XPS) were performed to gain a deeper understanding of the Gel/Zr/EGCG hydrogels interactions.

**Figure 3:**
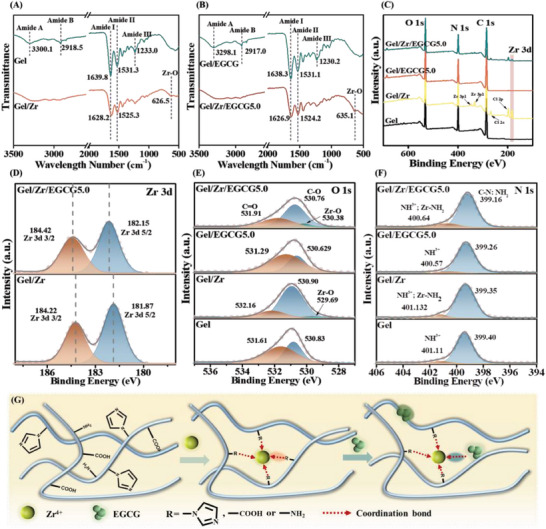
Interaction types within the Gel‐Zr‐EGCG hydrogel. FTIR spectra of A) Gel/Zr and B) Gel/Zr/EGCG5.0. C) XPS spectra of Gel, Gel/Zr, Gel/EGCG5.0, and Gel/Zr/EGCG5.0. XPS spectra of D) Zr 3d, E) N 1s, and F) O 1s of Gel, Gel/Zr, Gel/EGCG5.0, and Gel/Zr/EGCG5.0. G) Schematic representation of the possible interaction modes in Gel/Zr/EGCG hydrogels.

As shown in **Figure** **3**A,B, the characteristic absorption bands of Gel were observed at 1639.8 cm^−1^ (amide I, C═O group stretching), 1531.3 cm^−1^ (amide II, C═O group stretching, N─H group bending, and C═N group stretching vibrations), and 1233.0 cm^−1^ (amide III, in‐plane vibrations of amide‐binding C─N and N─H groups). The redshift of the amide II absorption bands of Gel/Zr and Gel/Zr/EGCG5.0 hydrogel compared with that of Gel (1531.3 cm^−1^) confirmed the formation of coordination bonds between Gel and the metal ions.^[^ ^29^, ^30^ ^]^ When Zr^4+^ coordinates with Gel, it rearranges the triple‐helix‐like structure of Gel, hydrogen bonding in Gel becomes easier to form. The carbonyl stretching vibrational peak of Gel at 1639.8 cm^−1^ and the Gel/Zr redshift to 1628.2 cm^−1^ confirms that the hydrogen bonding of Gel is strengthened. In addition, under the same conditions using bovine serum albumin (BSA) and lysozyme (Lys) to construct metal‐ligand hydrogels was found to be unable to form hydrogels. This indirectly confirms that hydrogen bonding within gelatin is also a non‐negligible force in the construction of Gel/Zr hydrogels (Figure S6, Supporting Information). In addition, the FTIR spectra of Gel/Zr and Gel/Zr/EGCG5.0 hydrogel included characteristic C─O peaks at 626.5 and 635.1 cm^−1^, respectively,^[^ ^31^ ^]^ which was attributed to the coordination of the carboxyl group of Gel with Zr^4+^. On adding EGCG to the hydrogels, the polyphenols initially interacted with the protein molecules through hydrophobic interactions and entered a hydrophobic pocket, followed by subsequent multipoint hydrogen bonding. Gel/EGCG5.0 also showed a red shift in the amide I and II bands compared with those of Gel, demonstrating the hydrogen bonding of EGCG with Gel.^[^ ^32^ ^]^ Gel and Gel/EGCG5.0 also included amide A bands at 3300.1 and 3298.1 cm^−1^, respectively. The vibration wave number of the N─H group was 3300–3500 cm^−1^. This position was shifted towards the lower frequencies due to the inclusion of the N─H group in a hydrogen bond within the peptide. The N─H group of Gel has been suggested to form a hydrogen bond with the ─OH group of EGCG.^[^ ^33^ ^]^

To further test this conjecture, we further explored the interactions between Gel, Zr^4+^, and EGCG through XPS. As shown in Figure 3C,D, the XPS spectra showed clear peaks corresponding to Zr 3d element in the Gel/Zr and Gel/Zr/EGCG5.0 hydrogel, indicating the successful capture of Zr 3d by Gel. The gradual shift in the characteristic Zr 3d_5/2_ and Zr 3d_3/2_ peaks (184.22 and 181.87.4 eV, respectively, for Gel/Zr hydrogel) towards higher energies (184.42 and 182.15 eV, respectively, for Gel/Zr/EGCG5.0 hydrogel) was attributed to an increase in the number of electrons supplied by Zr^4+^ in the Gel/Zr/EGCG5.0 hydrogel. This suggested strong coordination interactions between Zr^4+^ and EGCG.^[^ ^34^ ^]^ The metal phenolic networks (MPNs) formation was evidenced by the 100 nm nanoparticles formed upon direct mixing of Zr^4+^ with EGCG (Figure S7A,B, Supporting Information). More pronounced Tyndall phenomenon was observed in the Gel/Zr/EGCG5.0 hydrogel, indicating the presence of nanoparticles (Figure S7C, Supporting Information).

Due to the coordination interactions between Zr^4+^ and COOH in Gel, the C = O and C─O peaks in Gel/Zr and Gel/Zr/EGCG5.0 hydrogel were at higher binding energies compared with those of Gel and Gel/EGCG5.0 (Figure 3E). Importantly, the new peaks (Zr─O) appeared at 529.69 and 530.38 eV in Gel/Zr and Gel/Zr/EGCG5.0 hydrogel, respectively,^[^ ^35^ ^]^ with the area of Gel/Zr/EGCG5.0 hydrogel being 2.5% larger than that of Gel/Zr hydrogel, likely due to the metal–polyphenol interactions.^[^ ^36^, ^37^ ^]^ In addition, the C═O peak moved from 531.61 eV in Gel to 531.42 eV in Gel/EGCG5.0, indicating the formation of hydrogen bonds between Gel and EGCG.^[^ ^38^ ^]^ Similarly, the presence of hydrogen bonds (Figure 3F) was further confirmed by the lower binding energy shifts of C─N; NH_2_ N 1s for Gel/Zr and Gel/Zr/EGCG5.0 hydrogel compared with those of Gel and Gel/EGCG5.0. Additionally, the shift in the NH^3+^ peaks to higher binding energies in Gel/Zr and Gel/Zr/EGCG5.0 hydrogel compared with those of Gel and Gel/EGCG5.0 indicated the occurrence of metal coordination.^[^ ^39^ ^]^ The peak area ratios of the NH^3+^ in Gel/Zr and Gel/Zr/EGCG5.0 hydrogel increased by 2.26% and 6.56%, respectively, compared with those of Gel and Gel/EGCG5.0, which could be explained by the formation of some Zr═NH_2_ complexes that increased the peak areas.

Three major forces of Gel/Zr/EGCG hydrogels are illustrated in Figure 3G: 1) Coordination of Zr^4+^ with Gel side‐chain functional groups (such as amino and carboxylic acid groups) and consequently cross‐linking to form hydrogels, the main force in hydrogel formation. 2) Zr^4+^ coordinates with EGCG to form MPNs, accompanied by the formation of Zr^4+^/EGCG nanoparticles. 3) EGCG as an excellent hydrogen donor forms hydrogen bonds with Gel, and the addition of Zr^4+^ further increases the formation of hydrogen bonds within gelatin. The more detailed coordination chemistry is schematically illustrated in Figures S8 (Supporting Information).

### 2.3 In Vitro Biocompatibility and Intracellular Antioxidant Capability of Gel/Zr/EGCG1.0 Hydrogel

**Figure 4:**
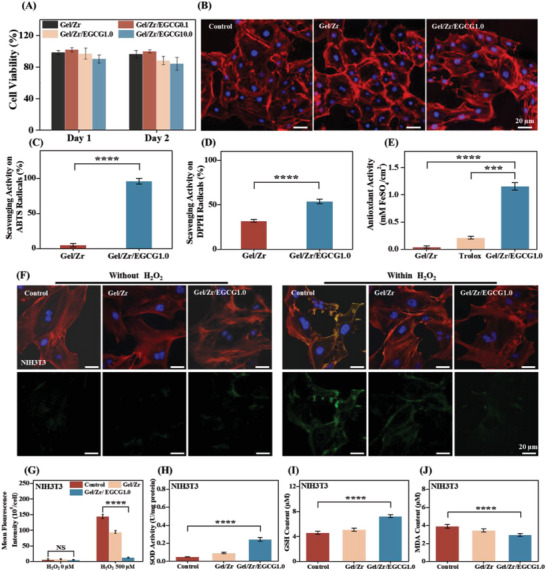
In vitro biocompatibility and intracellular antioxidant capability of Gel/Zr/EGCG1.0 hydrogel. A) The viability of NIH3T3 cells cultured in Gel/Zr and Gel/Zr/EGCG hydrogel extracts after one and 2 days, respectively (*n* = 5). B) Fluorescence images of NIH3T3 cells cocultured with the Gel/Zr/EGCG1.0 hydrogel for 2 d (The nucleus is blue and the cytoskeleton is red). C) ABTS scavenging capability of the Gel/Zr/EGCG1.0 hydrogel (*n* = 4). D) DPPH scavenging capability of the Gel/Zr/EGCG1.0 hydrogel (*n* = 4). E) Total antioxidant capacity of the Gel/Zr/EGCG1.0 hydrogel (*n* = 4). F) The intercellular ROS scavenging capability of the hydrogels was evaluated by DCFH‐DA (green fluorescence; left and right are before and after H_2_O_2_ stimulation, respectively). G) Fluorescence intensity statistics for DCFH‐DA (*n* = 4). H) SOD analysis with H_2_O_2_ stimulation (*n* = 4). I) GSH/GSSG analysis with H_2_O_2_ stimulation (*n* = 4). J) MDA analysis with H_2_O_2_ stimulation (*n* = 4).

Since hydrogel dressings are in direct contact with wounds, low cytotoxicity is a key factor determining their application prospects.^[^ ^40^, ^41^ ^]^ To evaluate the biocompatibility of the Gel/Zr/EGCG hydrogels, NIH3T3 and DC2.4 cells were cultured in hydrogel extracts and the cell viability was continuously monitored for two days. During the experiment, both cell types proliferated and diffused in the presence of the Gel/Zr and Gel/Zr/EGCG hydrogels, with almost identical growth rates, indicating that these hydrogels had no adverse effects on the cells (**Figure** **4**A and Figure S9A, Supporting Information). Meanwhile, the morphologies of the cells cultured with Gel/Zr and Gel/Zr/EGCG hydrogels were observed (Figure 4B and Figure S9B, Supporting Information). The data indicated that Gel/Zr/EGCG hydrogels are biocompatible and improve cell growth and viability in the presence of appropriate EGCG quantities. The Gel/Zr/EGCG1.0 hydrogel achieved a good balance between the overall properties and cytocompatibility. Thus, Gel/Zr/EGCG1.0 hydrogel was chosen as a representative example for further experimental evaluations.

The antioxidant capacity of the Gel/Zr/EGCG1.0 hydrogel was evaluated by DPPH and ABTS radical scavenging assays and found to be essential for suppressing oxidative stress in cells. The Gel/Zr hydrogel showed weak DPPH and ABTS scavenging abilities. Adding EGCG significantly enhanced the scavenging ability of the hydrogel (Figure 4C,D), with DPPH and ABTS radical scavenging rates of 97% and 56%, respectively, which were 19.4‐ and 1.7‐fold higher than those of the Gel/Zr hydrogel. A FRAP assay was used to determine the total antioxidant capacity for further verification. In the FRAP total antioxidant capacity test, Gel/Zr showed a weak antioxidant capacity, while the Gel/Zr/EGCG1.0 hydrogel showed significantly higher total antioxidant capacity than those of the Gel/Zr hydrogel and the positive control (1 × 10^−3^ m Trolox (vitamin E analog); EGCG hydrogel concentration approximately 0.01 × 10^−6^ m) (Figure 4E). In vitro antioxidant experiments showed that the Gel/Zr/EGCG1.0 hydrogel had an excellent antioxidant ability.

In the intracellular antioxidant assays, the fluorescent dye DCFH‐DA was used to assess intracellular ROS levels. DCFH‐DA can freely penetrate the cell membrane, after which it can be hydrolyzed to DCFH by intracellular esterase; however, the presence of ROS can cause nonfluorescent DCFH‐DA to generate green fluorescence. Therefore, oxidative stress was induced in NIH3T3 and DC2.4 cells by H_2_O_2_ and intracellular ROS generation was detected using DCFH‐DA. The control group of NIH3T3 and DC2.4 cells with no H_2_O_2_ induction showed no obvious green fluorescence and no significant difference, indicating the absence of significant amounts of ROS in normal cultured cells and material cultured cells. In H_2_O_2_ ‐induced NIH3T3 and DC2.4 cells, intense green fluorescence was observed, indicating an excess of intracellular ROS production (Figure 4F and Figure S9C, Supporting Information). The Gel/Zr hydrogel partially reduced the fluorescence intensity, which was further reduced by the Gel/Zr/EGCG hydrogels (Figure 4G and Figure S9D, Supporting Information). The mean fluorescence intensity decreased in the order of Control > Gel/Zr > Gel/Zr/EGCG1.0 group. These results showed that introducing EGCG into the hydrogel could effectively eliminate the generation of intracellular ROS.

To evaluate the intracellular ROS clearance mechanisms of hydrogels, we investigated the expressions of three cellular markers of oxidative stress, namely, SOD, GSH/GSSG, and MDA. SOD catalyzes the disproportionation of superoxide anions to produce H_2_O_2_ and O_2_ and is an important antioxidant enzyme in vivo. GSH is a major source of sulfhydryl groups in most living cells, is important for maintaining the proper redox states of protein sulfhydryl groups and is the key antioxidant in animal cells. Following H_2_O_2_ stimulation, the SOD and GSH levels in the Gel/Zr/EGCG1.0 hydrogel treatment group were significantly upregulated compared with those of the control group and Gel/Zr (Figure 4H–I and Figure S9E,F, Supporting Information), indicating that EGCG maintains the stability of the SOD and GSH levels by rapidly removing excess ROS. MDA, a natural product of lipid oxidation, is an important indicator of cellular oxidative stress damage. The MDA results shown in Figure 4J and Figure S9G (Supporting Information) show an upregulation of MDA levels in H_2_O_2_‐stimulated cells that are effectively controlled by Gel/Zr/EGCG1.0, indicating that the oxidative stress response was significantly inhibited. In conclusion, the Gel/Zr/EGCG1.0 hydrogel has great potential for protecting cells and tissues against damage in the high‐ROS microenvironments of diabetic wounds. These results suggest the good antioxidant activity of Gel/Zr/EGCG1.0 hydrogel, which can handle the sudden increase and persistence of ROS during tissue epithelialization repair.

### 2.4 Cell Survival and Migration under H_2_O_2_, RT‐qPCR Testing

**Figure 5:**
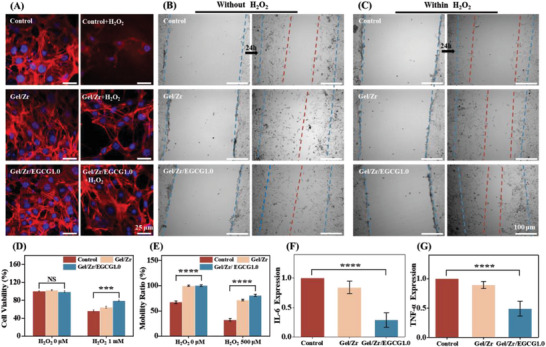
Cell survival and migration under H_2_O_2_. A) Cytoskeletal structure of the NIH3T3 cells after incubation in the solution containing hydrogels and H_2_O_2_. B) Migration effect of the NIH3T3 cells before and after coculturing with hydrogels for 24 h. C) A rendering of NIH3T3 cell migration before and after coculturing them with the hydrogel for 24 h in the presence of H_2_O_2_ (the blue and red dotted lines represent the scratch positions before and 24 h after NIH3T3 cell migration, respectively). D) NIH3T3 cells survival rate measured by CCK8 kit after incubation in the solution containing hydrogels and H_2_O_2_ (*n* = 3). E) Histogram of NIH3T3 cell migration rates. F,G) RNA expression of IL‐6 and TNF‐α.

Treatments with high‐strength H_2_O_2_ for 8 h induced oxidative stress injuries in NIH3T3 and DC2.4 cells. As shown in **Figure** **5**A and Figure S10A (Supporting Information), while the cellular morphologies of both the control and Gel/Zr cell groups showed some damage, the protective effect of EGCG in the Gel/Zr/EGCG1.0 hydrogel group on scavenging ROS resulted in a morphology that was consistent with cells before H_2_O_2_ stimulation. Simultaneously, the CCK‐8 kit was used to evaluate the cell survival (Figure 5D and Figure S10B, Supporting Information). The Gel/Zr/EGCG1.0 hydrogel group reduced the oxidative stress damage of both NIH3T3 and DC2.4 cells and improved cell activity. We evaluated the potential of the Gel/Zr/EGCG1.0 hydrogel for promoting fibroblast migration by in vitro scratch healing experiments. Wound scratches were created in NIH3T3 cell monolayers and the changes in cell gaps of the scratches over time were measured to evaluate the migration. As shown in Figure 5B,C, 24 h after the scratch treatment, the cell gap in the Gel/Zr/EGCG1.0 hydrogel group was narrower than that of the control group, with the migration rates and densities decreasing in the order of Gel/Zr/EGCG1.0 > Gel/Zr > Control group. Although the cell migration rate of each group was affected by H_2_O_2_ addition, the Gel/Zr/EGCG1.0 hydrogel group maintained a good cell migration rate under oxidative stress (Figure 5E). Usually, excessive ROS in diabetic wounds trigger an inflammatory response that seriously affects wound healing. EGCG has excellent anti‐inflammatory properties. Therefore, we first induced inflammations in RAW cells with LPS, then treated them with Gel/Zr/EGCG1.0 hydrogels and evaluated the ability of the Gel/Zr/EGCG1.0 hydrogel to improve the inflammatory response (IL‐6 and IL‐10) by RT‐qPCR. The IL‐6 levels were downregulated by at least 70% and TNF‐α levels by at least 60% in the Gel/Zr/EGCG1.0 hydrogel group (Figure 5F,G). This strongly suggested the effectiveness of the Gel/Zr/EGCG1.0 hydrogel platform in reducing the inflammatory response. These results indicate that the Gel/Zr/EGCG1.0 hydrogel platform has great potential for promoting cells.

### 2.5 Quantification of EGCG and Zr^4+^ Release from Hydrogel and In Vitro Antibacterial Properties

The release of EGCG and Zr^4+^ from the hydrogel was examined by UV–visible spectrophotometry (UV–Vis) and inductively coupled plasma mass spectrometry (ICP‐MS), respectively. The release of EGCG, as a small‐molecule drug, was faster, and it could be released up to 60% by the third day (Figure S11A, Supporting Information), whereas the release of Zr^4+^ is slowed down because the Zr^4+^ is re‐captured by the reactive groups of the gelatin during the release process (Figure S11B, Supporting Information).

An ideal diabetic wound dressing should have good antibacterial properties to reduce the number of bacteria in the wound, reduce inflammation, and promote wound healing. When the Zr^4+^ ions in the hydrogel contact with the bacterial membrane, its integrity is destroyed and the contents leak out, which interferes with the metabolic activities of the cell, resulting in bacterial death. The plant polyphenol EGCG destroys the integrity of the cell walls and permeability of the cell membranes and exhibits antibacterial effects by inhibiting biological macromolecular synthesis. MPNs are an emerging organic‐inorganic hybrid network system that has been gradually developed in recent years, utilizing phenol ligands coordinated with metal ions to exhibit excellent multifunctional properties such as anti‐inflammatory, antioxidant, and antibacterial properties.^[^ ^42^, ^43^ ^]^ The inhibition zone test verifies the lowest antibacterial concentration of Zr^4+^ and Zr^4+^/EGCG (Figure S11C, Supporting Information). In the gradient antimicrobial experiment of Zr^4+^, it can be found that with the decrease of Zr^4+^ concentration, the area of the inhibition circle decreases, and the inhibition zone disappears at the concentration of 0.3 × 10^−3^ m, which indicates that the lowest inhibition concentration of Zr^4+^ is 0.3 × 10^−3^ m. In addition, in the inhibition circle experiments of EGCG alone, only 2 mg mL^−1^ of EGCG can form a very fuzzy inhibition circle, which indicates the inhibition ability of EGCG what is limited and cannot be relied on its inhibition capacity alone. Surprisingly, in the Zr^4+^/EGCG zone of inhibition experiments, the zone of inhibition at 12.5 × 10^−3^ m and 0.6 × 10^−3^ m of Zr^4+^/EGC were obviously larger than that of Zr^4+^, and clear circles of inhibition still existed at 0.3 × 10^−3^ m of Zr^4+^/EGCG, which directly demonstrated that the combination of Zr^4+^/EGCG could enhance the antimicrobial effect.

In the release experiment of EGCG and Zr^4+^ it can be found that EGCG is released rapidly, which has a good effect on the timely clearance of ROS from the wound. In addition, the slow release of Zr^4+^ can reduce the toxicity of metal ions to the organism, while the synergistic effect of low concentration of Zr^4+^ and high concentration of EGCG can bring excellent antibacterial effect.

The antibacterial rates of Gel, Gel/Zr, and Gel/Zr/EGCG1.0 group against *E. coli* and *S. aureus* were evaluated by spectrophotometry and surface antibacterial experiments. Plate counting demonstrated that the Gel/Zr hydrogel was very effective against *E. coli* and *S. aureus*, since the colonies on the agar plate were significantly fewer than those of the control group. After adding EGCG, the colonies on the agar plate disappeared, it was also demonstrated that EGCG and Zr^4+^ had a combined antimicrobial effect (Figure S12A,B, Supporting Information). Statistical histograms of the colonies are shown in Figure S12C (Supporting Information). Simultaneously, we co‐cultured the bacteria with the hydrogel for 6 h and spectrophotometry showed the elimination of more than 90% of *E. coli* and *S. aureus* (Figure S12D, Supporting Information). The results show the clear antibacterial activity of the Gel/Zr hydrogel against *E. coli* and *S. aureus*. Adding polyphenols promoted the antibacterial activity of the hydrogel and synergistically affected the antibacterial activity of the Gel/Zr hydrogel. The Gel/Zr/EGCG1.0 hydrogel possessed excellent antibacterial activity and we believe that it has great potential for the effective prevention and treatment of wound bacterial infections.

### 2.6 Promoting Wound Healing Performance of the Gel/Zr/EGCG1.0 Hydrogel

**Figure 6:**
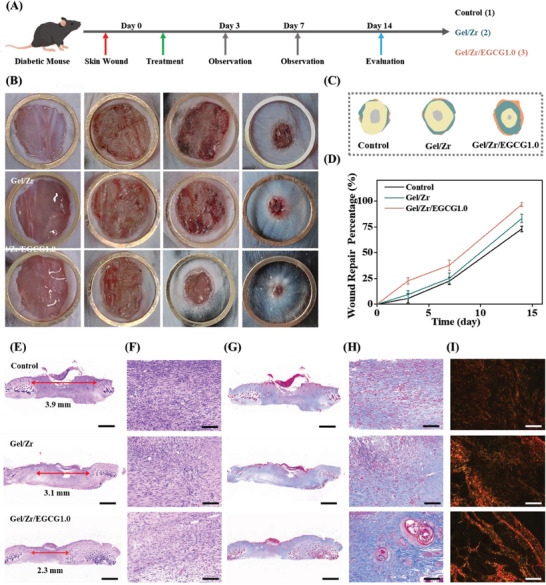
Wound healing and histopathological images of wound tissues in diabetic mice. A) Schematic illustration for chronic wound generation and the subsequent healing process. B) Representative photographs of chronic wound healing on days 0, 3, 7, and 14 (*n* = 3). C) Wound fractions healed by different treatments on days 0, 3, 7, and 14 (*n* = 3). D) Rate of wound recovery during chronic wound healing (*n* = 3). E) H&E staining of the wound area on day 14 (*n* = 3, scale bar = 1 mm). F) Partially enlarged H&E images (scale bar = 100 µm). G) Masson's staining of the wound area on day 14 (*n* = 3, scale bar = 1 mm). H) Partially enlarged Masson images (scale bar = 100 µm). I) Representative scarlet staining sections PS red (*n* = 3, scale bar = 50 µm).

The effects of the Gel/Zr/EGCG1.0 hydrogel on repairing acute skin models and chronic diabetic skin in mice were studied. **Figure** **6**A and Figure S13A (Supporting Information) show the treatment process of acute and chronic skin wounds, respectively; Figure 6B and Figure S13B (Supporting Information) show macroscopic views of the wounds after 3, 7, and 14 days of Gel/Zr and Gel/Zr/EGCG1.0 hydrogel treatments for two groups of different models. As shown in Figure 6C,D, and Figure S13C,D (Supporting Information) the wound areas of the acute and chronic models were evaluated from days 0 to 14. The wound areas decreased with increasing healing times. For the same healing time, the wound areas of the control, Gel/Zr, and Gel/Zr/EGCG1.0 hydrogel groups decreased successively, indicating that the addition of EGCG in the Gel/Zr/EGCG1.0 hydrogel enhanced the wound healing ability.

The morphologies of diabetic wounds were evaluated by H&E and Masson's staining. As shown in Figure 6E,F and Figure S13E (Supporting Information), the Gel/Zr/EGCG1.0 hydrogel group exhibited the highest epithelialization rate, suggesting the efficient removal of ROS in the wound microenvironment to mitigate cellular oxidative damage and facilitating growth factor secretion. Notably, collagen deposition in the Gel/Zr/EGCG group was superior to those of the Gel/Zr and control groups, as indicated by Masson's staining (Figure 6G,H and Figure S13F, Supporting Information). The Gel/Zr/EGCG1.0 hydrogel group showed blue deposition, while red was almost invisible, indicating that the Gel/Zr/EGCG1.0 hydrogel group had more collagen deposited on the wound surface. PS staining was used to further demonstrate the maturation of wound collagen and assess the degree of collagen deposition. The characteristic color of collagen during PS staining enables the differentiation between various types under polarized light; for instance, type III collagen in the early stages of wound healing appears as greenish‐yellow while mature type I collagen in the late stage is orange‐red. As shown in Figure 6I and Figure S13G (Supporting Information), the center of the control group had almost no collagen deposition on the 14 day, while type III collagen was mainly deposited in the initial stages of wound healing. The Gel/Zr/EGCG1.0 hydrogel group had more collagen deposition, which was mainly orange‐colored type I collagen. These results confirmed that Gel/Zr/EGCG1.0 hydrogel promote collagen maturation during wound healing. The results showed that Gel/Zr/EGCG1.0 hydrogel promote wound healing mainly because of their positive antioxidant activities, which effectively remove excess ROS to reduce oxidative damage to newly formed wound granulation tissues and blood vessels.

### 2.7 Immunohistochemical Analysis

**Figure 7:**
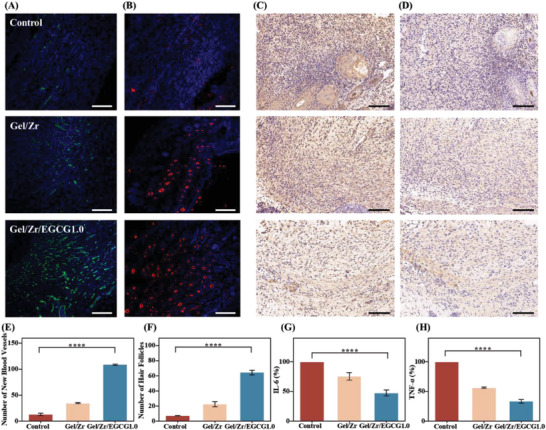
Immunohistochemical staining of wound tissues on day 14. A–D) Representative staining images of CD31, CK19, IL‐6, and TNF‐α at the wound site (*n* = 3, scale bar = 100 µm). E–H) Statistical analyses of CD31, CK19, IL‐6, and TNF‐α (*n* = 3).

Immunohistochemistry was used to detect the healing sites of chronic diabetic wounds and the expression of CD31, CK19, IL‐6, and TNF‐α on day 14. We selected CD31 and CK19 for immunofluorescence staining analysis since they represent the levels of angiogenesis and hair follicle regeneration in the wound, respectively, and their expression levels were significantly increased in the Gel/Zr/EGCG1.0 hydrogel group (**Figure** **7**A,B,E,F).

The results suggest that the hydrogel effectively promotes wound hair follicle regeneration and angiogenesis. In general, IL‐6 and TNF‐α are involved in the pathogenesis of systemic inflammatory response syndrome. The control group showed severe inflammation on newly formed skin tissue and quantitative analysis clearly showed that the Gel/Zr/EGCG1.0 hydrogel treatment had a better effect on re‐epithelialization and the inhibition of IL‐6 and TNF‐α expression (Figure 7C,D,G,H). These results indicate that Gel/Zr/EGCG1.0 hydrogel can reduce the inflammatory levels of wounds under high ROS conditions and promote angiogenesis to accelerate diabetic wound healing.

### 2.8 In Vivo Biocompatibility of Gel/Zr/EGCG1.0 Hydrogel

**Figure 8:**
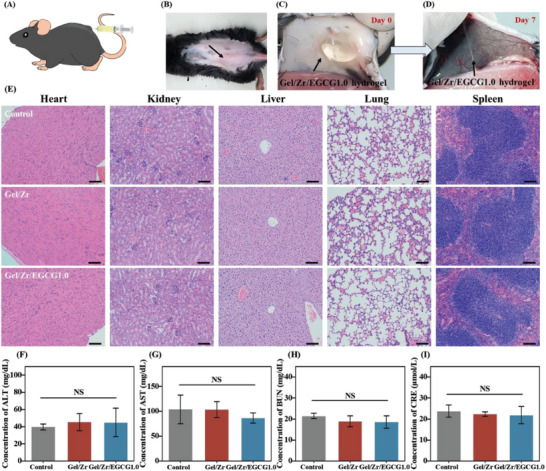
In vivo biocompatibility of Gel/Zr/EGCG1.0 hydrogel. A) Schematic of the subcutaneous injection of hydrogel. Photos of B) the back of mice after just receiving a subcutaneous injection of the Gel/Zr/EGCG1.0 hydrogel, and the subcutaneous Gel/Zr/EGCG1.0 hydrogel after injection at C) day 0 and D) day 7. E) H&E staining of major mice organs (*n* = 3, scale bar = 100 µm). The serum liver function indicators were: F) ALT and G) AST (*n* = 3). The serum renal function indicators were: H) BUN and I) CRE (*n* = 3).

To further evaluate the biosafety of our hydrogel, as shown in **Figure** **8**A,B, approximately 100 µL of the Gel/Zr/EGCG1.0 hydrogel was injected into the mice subcutaneously. After sacrificing the mice at days 0 and 7, their skin was sectioned beneath the hydrogels, observed, and photographed (Figure 8C,D). After euthanizing the mice, the injection sites were cut open and the hydrogel appeared to have been mostly degraded (Figure 8D). Simultaneously, histopathological examinations of the mice showed that the hearts, livers, spleens, lungs, and kidneys in the experimental group were not significantly different from those in the control group, with no obvious lesions observed (Figure 8E).

The serum biochemical analysis results are shown in terms of ALT and AST, which are two important indicators of liver function. No significant difference was observed between the Gel/Zr/EGCG1.0 hydrogel and control groups Figure 8F,G. The functional indexes BUN and CRE of the Gel/Zr/EGCG1.0 hydrogel treatment group were not significantly different from those of the control group, indicating that Gel/Zr/EGCG1.0 hydrogel does no damage to the kidneys (Figure 8H,I). In general, the Gel/Zr/EGCG1.0 hydrogel has degradability and high biocompatibility, which indicates its excellent potential in tissue repair.

## 3 Discussion

Proteins are promising construction units for functional hydrogel materials because of their potent functions and ordered spatial structures. Protein‐based hydrogels are mainly prepared by two kinds of methods, 1) direct cross‐linking by cross‐linking agents, such as EDC/NHS, tetrakis (hydroxymethyl) phosphonium sulfate (THPS), etc., and 2) chemical modification of cross‐linkable groups and post‐cross‐linking. All these strategies have shortcomings that make them difficult to be used in clinics, such as the toxicity of the chemical reagents used for crosslinking and modification, the complexity of chemical modification and post‐crosslinking processes, and the time‐consuming purification. The strategy we reported here represent a simple and efficient to prepare Gel hydrogels by directly mixing of Zr ions and Gel. In this hydrogel, Zr ion is as a cross‐linking agent to form the hydrogel by coordination interaction with the active functional groups on Gel, such as Zr─O and Zr─NH_2_. Considering that amino and carboxyl groups are abundant residues present in most proteins, this strategy is prospective to be extended to construct other protein hydrogels.

The microenvironment with high ROS level is a typical feature of diabetic wounds. EGCG, a green tea extract, has excellent antioxidant and anti‐inflammatory properties, which are helpful to remodel the microenvironment of diabetic wounds. In addition, EGCG alleviates intracellular inflammation, significantly reducing the expression of pro‐inflammatory factors, is beneficial for the wound repair. Besides, EGCG has strong chelating ability to metal ions through the catechol groups and binding ability to protein through multiply interactions, such as hydrogen bonding, hydrophobic interactions, etc. The introduction of EGCG into Gel/Zr hydrogel make the shear thinning behavior becomes more pronounced, with improved injection properties. Meanwhile, Gel/Zr/EGCG hydrogel contains rich catechol groups, which can promote the adhesion of the hydrogel to the substrate, enabling better use of the hydrogel for wound repair. In chronic wound repair treatment, Gel/Zr/EGCG hydrogel adheres to the damaged wound, imitates the structure of natural ECM, promotes cell adhesion and growth, and provides a microenvironment adapted to skin repair. After 14 days of treatment, it effectively promoted wound recovery, re‐epithelialization, collagen deposition, and angiogenesis.

In this study, when cells were cultured in the leachate of the hydrogels, no apparent cytotoxicity was observed, and further in vivo safety evaluation was verified by subcutaneous embedding of the hydrogels in mice. The hydrogel was degraded completely by day 7, and the H&E sections of the heart, liver, spleen, lung, and kidney showed that the hydrogel did not cause significant damage to the internal organs of the mice. In addition, the evaluation of the liver and kidney function indexes of blood were within the normal range. The non‐detectable cytotoxicity might be attributed to two main reasons: 1) Zr metal is commonly used for in vivo implantation of medical alloy materials due to its biologically inert and good biocompatibility. 2), with the addition of EGCG being able to further coordinate with the free Zr ions in the hydrogel, this would largely avoid the explosive release of Zr ions. Although there was no detectable cytotoxicity, the long‐term biosafety of Gel/Zr/EGCG hydrogel was definitely necessary for further clinical trial.

The injectable Gel/Zr/EGCG hydrogel allows the hydrogel to fill in irregular wounds or gaps between desired tissue sites and enables a self‐healing function in the event of hydrogel fracture caused by movement. The adhesion of Gel/Zr/EGCG hydrogel to skin make it well sealed the wounds and avoid contacting external pathogens. In the in vivo test, Gel/Zr/EGCG hydrogel can degrade in weeks and slowly during the wound healing, thus largely avoiding secondary damage caused by hydrogel removal. Considering these above‐mentioned merits of Gel/Zr/EGCG hydrogel provides a unique microenvironment in skin tissue repair, we believe that it has a promising future in wound repair.

In summary, an injectable, self‐healing, and adhesive Gel‐based hydrogel was successfully engineered using a simple and facile strategy, namely, the direct mixing of metal ions and Gel, which relies on the pH and the metal ions and Gel concentrations. In the hydrogel, the metal ions dynamically crosslinked the Gel through coordinative interactions between the rich Gel residual groups (such as carboxyl and amino groups) and Fe^3+^ or Zr^4+^. EGCG doping greatly improved the mechanical profiles (injectable, self‐healing, and adhesive properties) and biological functions (antioxidant and antibacterial properties) of the Gel/Zr/EGCG hydrogels. Both in vitro and in vivo tests demonstrated that the Gel/Zr/EGCG1.0 hydrogel exhibited fast cell migration, strong anti‐inflammation, and excellent antioxidant behavior, which could greatly remodel the microenvironments of diabetic chronic wounds and accelerate the healing rates of diabetic chronic wounds. In an in vivo biosafety test, the subcutaneously injected Gel/Zr/EGCG1.0 hydrogel degraded almost entirely within a week, with no detectable cytotoxicity. Considering the advantages of using Gel (multiple sources, excellent biocompatibility, and bioactivity) and the feasibility of this strategy (straightforward mixing with no chemical modifications), we believe that hydrogels have great potentials in various biomedical applications such as skin tissue engineering.

## 4 Experimental Section

### Materials

Gel (strength of the gelatin, approximately 100 g Bloom), sodium citrate (98%), citric acid monohydrate (99.5%), zirconium (IV) chloride (ZrCl_4_, 98%), and phosphate buffered solution (PBS, pH 7.2‐7.4) were purchased from Aladdin Industrial Corporation (Shanghai, China). Epigallocatechin gallate EGCG (95%), chloral hydrate (>99%), and streptozotocin (STZ, 98%) were purchased from Macklin (Shanghai, China). A cell counting Kit‐8 (CCK‐8) was procured from Dojindo Molecular Technologies (Shanghai, China). 4′,6‐Diamidino‐2‐phenylindole (DAPI), lipid peroxidation malondialdehyde (MDA) assay kit, GSH and GSSG assay Kit, total superoxide dismutase (SOD) assay kit with water‐soluble tetrazolium‐8 (WST‐8), and total antioxidant capacity assay kit with the ferric reducing ability of plasma (FRAP) method were purchased from the Beyotime Institute of Biotechnology (Shanghai, China). Hydrogen peroxide (H_2_O_2_), TRITC‐phalloidin, paraformaldehyde (4%), 2,2‐diphenyl‐1‐picrylhydrazyl (DPPH), 2,7‐dichlorodihydrofluorescein diacetate (DCFH‐DA), and 2,2′‐azino‐bis (3‐ethylbenzothiazoline‐6‐sulfonic acid) (ABTS) free radical scavenging capacity detection kit were purchased from Solarbio Life Science (Shanghai, China). Lipopolysaccharide (LPS) was purchased from Sigma‐Aldrich (Shanghai, China). RNA extraction, reverse transcription kit, and fluorescent dye SYBR Green were purchased from Vazyme (Nanjing, China). Fetal bovine serum, penicillin–streptomycin, and Dulbecco's modified Eagle's medium (DMEM) were obtained from Gibco (USA). Round glass coverslips 14 mm in diameter were cleaned with piranha solution and stored in 75% ethyl alcohol.

### Experimental Design

To obtain the phase diagrams of Gel mixtures with different metal ions, the concentration of Gel was determined by first adding 8 µL of 0‐1 m ZrCl_4_ to 92 µL of a 0%–10% (w/v) Gel solution. Next, 10% Gel (w/v) was mixed with ZrCl_4_, FeCl_3_, and CuCl_2_ at concentrations of 0–1 m, and the pH was adjusted from 0–10.0. The Gel/Zr hydrogel was formed by adding 8 µL of a 1 m ZrCl_4_ solution to 92 µL of a 10% Gel solution. The final concentrations of ZrCl_4_ and Gel in the hydrogel were 80 × 10^−3^ m and 10% (w/v), respectively. To prepare the Gel/Zr/EGCG hydrogels, Gel was dissolved in an EGCG solution at different concentrations, with the same subsequent experimental steps as those of the Gel/Zr hydrogel. (The contents of EGCG in the hydrogel are 0.1, 1.0, 5.0, and 10.0 mg/mL for Gel/Zr/EGCG0.1, Gel/Zr/EGCG1.0, Gel/Zr/EGCG5.0, and Gel/Zr/EGCG10.0, respectively).

### Injectable and Self‐Healing Properties of Gel/Zr/EGCG Hydrogels

To examine the injectability of the Gel/Zr/EGCG hydrogels, Gel/Zr/EGCG1.0 hydrogel was extruded from a 2 mm needle with a 2.5 mL syringe to produce a specific shape. For the macroscopic examination of the self‐healing properties of the hydrogel, we exposed rhodamine B‐stained and unstained hydrogels to each other for 20 min at room temperature before stretching them with forceps to demonstrate the degree of healing of both hydrogels. The injectable and self‐healing processes of the hydrogel were photographed and recorded.

### Rheological Testing

Rheological measurements were performed using a discovery hybrid rheometer (DHR‐2; TA Instruments, UK) equipped with an 8‐mm diameter plate. The storage (*G*′) and loss (*G*″) moduli of the hydrogel were measured at a 1% constant strain and oscillation frequency of 0.1–100 rad s^−1^ at 25 °C. Strain scanning tests were performed on molded specimens at 37 °C to verify the linear viscoelastic region and the linear viscoelastic region with oscillatory strains increasing from 0.1% to 1000%. The viscosity behavior of the hydrogel was then assessed using a shear thinning test with a shear rate adjusted from 0.1 to 60 rad s^−1^.^[^ ^44^ ^]^

### Hydrogel Characterization

The hydrogel was lyophilized and coated with platinum using a high vacuum sputter coater (EM ACE600, Leica, Germany) to improve its electrical conductivity. The morphology of the hydrogel was then observed by field emission scanning electron microscopy (SEM, Hitachi, Japan) at 5 keV. To determine the swelling rate of the hydrogel in vitro, the lyophilized hydrogel was weighed and placed into PBS. At different time points, the lyophilized hydrogel was removed from the PBS and weighed after drying with filter paper. The swelling rate was calculated using the following formula:

(1) $$S\%=\left[\left(Wa-Wb\right)/Wb\right]\times 100\%$$

where *W*b is the mass of the lyophilized gel before soaking and *W*a is its mass after soaking for a certain duration.^[^ ^45^ ^]^

### Adhesive Performance Test of the Hydrogel

Fresh porcine skin was used to study the ability of the hydrogel to adhere to human skin.^[^ ^46^ ^]^ Briefly, the fresh porcine skin was first cut into 20 mm × 80 mm slices. Next, 100 mg of the hydrogel was evenly applied to the porcine skin dermis and covered with another piece of porcine skin dermis; the adhesive area of the porcine skin was 20 mm × 20 mm. The adhered pigskin was subjected to a pressure of 0.1 N for 12 h in ambient conditions and covered with PBS‐soaked gauze to retain its moisture and achieve a relatively stable state between the hydrogel and the pig skin. An electronic universal material testing machine (UTM, Instron Frame model 5944) equipped with a 100 N load cell was used to examine the maximum adhesion strength of the hydrogel to the porcine skin. Each experiment was repeated at least five times and the results were reported as the mean ± standard deviation (SD).

### Quantification of Zr^4+^ Release from Hydrogel

To quantify the release of Zr^4+^ from Gel/Zr/EGCG1.0 hydrogels, the hydrogels were incubated in 1 mL of Zr‐free pH 5.0 and pH 7.0 buffers and maintained at 37 °C. All PBS buffers were collected at each preset time point and replenished with the same volume of fresh buffer. All hydrogels were analyzed in triplicate. The release of Zr was determined by ICP‐MS (Agilent, USA).

### Quantification of EGCG Release from Hydrogel

The hydrogels loaded with EGCG were completely immersed in phosphate buffer solutions of pH 5.0 and pH 7.0 and placed in a 37 °C incubator and shaken at a rate of 100 rpm. At a predetermined time, 1 mL of buffer was removed, and the same volume of buffer was added at 37 °C. The EGCG content of the samples was measured at 274 nm by UV–Vis (Cary 5000 UV–visible–NIR spectrophotometer).

### Antibacterial Performance of the Hydrogel

The antibacterial performance of the Gel/Zr/EGCG hydrogels was evaluated by its antibacterial activity against *Escherichia coli* and *Staphylococcus aureus*. *E. coli* and *S. aureus* were inoculated into a sterilized Luria‐Bertani (LB) liquid medium and placed overnight in a 180 rpm shaker to achieve the desired concentration. Briefly, 100 µL of hydrogel was injected into 24‐well cell culture plates, followed by 20 µL of the bacterial solution (1 × 10^6^ CFU mL^−1^). The control was an LB medium containing only bacteria. The 24‐well plates were incubated in a shaker at 180 rpm for 6 h at 37 °C, and the supernatants from the plates were diluted 10^4^ times. The diluted bacterial solutions were evenly coated on LB agar plates and incubated at 37 °C for 16 h. Images were then recorded and the colonies on the plates were counted.^[^ ^47^ ^]^

The results were expressed in terms of the inhibition rate (%): Inhibition rate (%) = (number of colonies in the control group‐number of colonies in the experimental group) / number of colonies in the control group × 100% for the control and sample groups, five parallel experiments were conducted.

Spectrophotometry was also used to examine the antibacterial effect. First, 100 µL of the hydrogel sample was injected into a 4 mL Eppendorf tube, to which 2 mL of LB liquid medium containing bacteria (5 × 10^5^ CFU mL^−1^) was added. LB medium containing only bacteria was used as a control. The plates were then incubated at 37 °C and 220 rpm for 6 h. The optical density at 600 nm (OD_600_) of the bacterial solution was measured using a spectrophotometer (1530, Thermo Fisher, Finland).

The results were expressed in terms of the inhibition rate (%): Inhibition rate (%) = (positive control OD_600_ value – sample groups OD_600_ value)/ (positive control OD_600_ – negative control OD_600_) × 100% for the control and sample groups, five parallel experiments were conducted.

Antibacterial sensitivity with agar diffusion test: Inoculate by applying an equal amount of suspension containing *Staphylococcus aureus* to the surface of the agar. Then, different concentrations of Zr^4+^, EGCG, and Zr^4+^/EGCG solutions were added dropwise to circular filter paper sheets, which were placed on the agar surface and incubated at 37 °C for 18 h.

### Cytotoxicity

For the CCK‐8 assays, dendritic cells DC2.4 cells and NIH3T3 cells were cultured with 10% FBS and 1% penicillin–streptomycin. The hydrogel (1 mg) was immersed in fresh DMEM medium (10 mL) at 37 °C for 24 h and passed through a 0.22 µm filter to prepare fresh hydrogel extracts for the cell experiments. First, 7 × 10^3^ cells were added to a 96‐well plate and the complete medium was replaced with an aqueous bleaching gel and leaching solution. After 24 and 48 h, the cells were incubated with a new medium containing 10% CCK‐8 for 4 h in the dark and the OD_450_ value was measured using a spectrophotometer. The cell viability was calculated using the following equation:

(2) $$\mathrm{Cell}\;\mathrm{viability}=C_{1}/C_{0}\times 100\%$$

where *C*_0_ and *C*_1_ are the OD_450_ of the control and experimental groups, respectively.^[^ ^48^ ^]^

The cytoskeletons and nuclei were stained with TRITC‐phalloidin and DAPI, respectively. Observations were made by confocal laser scanning microscopy (STELLARIS 5, Leica, Germany). Five parallel experiments were performed in the control and sample groups.

### Antioxidant Capacity of Hydrogels

The antioxidant capacity of the hydrogel was evaluated using total antioxidant capacity, ABTS radical, and DPPH radical scavenging assays in vitro. The FRAP method was used to determine the total antioxidant capacity of the system.^[^ ^7^ ^]^ Following the total antioxidant capacity assay, 10 µL of the hydrogel was added to a 48‐well plate followed by 360 µL of the FRAP working solution, which was then gently and evenly mixed and incubated for 37 min. Next, 185 µL of the mixture was removed, the absorbance was measured at 593 nm, and the total antioxidant capacity of the hydrogel was calculated. For the ABTS radical scavenging ability assay, 50 µL of the hydrogel was placed in 1 mL of an ABTS extract, ground to a fine level, and the supernatant extracted, mixed thoroughly with the ABTS free radical solution, left at room temperature for 6 min in the dark, and the absorbance measured at 405 nm. The ABTS radical scavenging capacity was then calculated. For the DPPH radical scavenging capacity assay, the hydrogel was added to a DPPH radical extract and ground, following which 25 µL of the supernatant was centrifuged (10 000 rpm) and 975 µL of the DPPH radical working solution was added. After mixing, the hydrogel was left at room temperature for 30 min in the dark, the absorbance was measured at 515 nm, and the DPPH radical scavenging rate was calculated. All experiments were performed five times and the results were expressed as the mean value.

Through changes in the cell morphology and cell number as well as the CCK‐8 cell activity, the antioxidant activity of the hydrogel in the presence of an excess of ROS in the cellular environment was tested. DC2.4 and NIH3T3 cells were added to 24‐well plates (2 × 10^4^ cells per well). The DC2.4 and NIH3T3 cells were induced with 1 × 10^−3^ m H_2_O for 8 h. After removing the culture media, the cells were fixed with 4% cell fixation solution (polyoxymethylene, POM), the cytomembranes were stained with TRITC‐phalloidin, and the nuclei were stained with DAPI. The cell morphology was observed, and the cell numbers were counted by confocal laser scanning microscope (A1, Nikon, Japan). Cell activity was detected at 450 nm using a CCK‐8 kit.

The anti‐oxidative ability of hydrogel in cells was detected using the fluorescent probe 2′,7′‐dichlorofluorescein diacetate (DCFH‐DA).^[^ ^49^ ^]^ The cells were cultured with the hydrogel extract for 24 h and induced with 500 × 10^−6^ m of H_2_O_2_ for 12 h. The medium was then removed, DCFH‐DA was added, and the cells were preserved for 20 min in a cell culture box at 37 °C. In a dark environment, the cells were immobilized with a 4% cell fixation solution (polyoxymethylene, POM), the cell membrane actin was stained with TRITC‐phalloidin, and the nuclei were stained with DAPI. The cells were photographed using confocal microscopy and the average fluorescence intensity was analyzed.

The antioxidant activity of the hydrogels in the presence of ROS was determined by measuring the intracellular total SOD, GSH, MDA,^[^ ^50^ ^]^ and ROS. The cells were cultured with the hydrogel extract for 24 h and induced with 500 × 10^−6^ m H_2_O_2_ for 12 h. First, the cells were collected with PBS at 4 °C and air‐conditioned. The homogenate was centrifuged at 4 °C and the supernatant was extracted as the sample to be tested. The supernatant was evenly mixed with WST‐8/enzyme and reaction start‐up working solutions at 37 °C for 30 min. The absorbance was measured at 450 nm. The total SOD activity in the cells was calculated. Second, the cells were collected with the protein removal reagent M solution and frozen and thawed twice. After 5 min in an ice bath, the supernatant was centrifugation at 10 000 g for 10 min to determine the total GSH. The supernatant and the total GSH detection solution were co‐incubated for 5 min at room temperature, and then a reduced nicotinamide adenine dinucleotide phosphate solution was added. After evenly mixing, the mixture was left to stand for 30 min and placed in a microplate reader at 412 nm. The GSH concentration was then calculated.^[^ ^3^ ^]^ Lastly, the cells were digested, collected with PBS, and lysed. After centrifugation at 1600 *g* for 10 min, the supernatant was taken, mixed with an MDA working solution, heated to 100 °C for 15 min, cooled to room temperature in a water bath, and the absorbance was measured at 532 nm. The MDA concentration was then calculated.

### In Vitro Scratch Wound Healing, Angiogenesis, and Quantitative Reverse Transcription Polymerase Chain Reaction (RT‐qPCR) Tests

Next, cell migration was performed. For the cell migration assay, NIH3T3 cells were first cultured in six‐well plates. The healthy cells at the bottom of the plates were scratched vertically with a pipetting gun and the hydrogel eluate without FBS was added after clearing the cell remnants with PBS. After culturing for 48 h, the cells were observed under an in situ microscope, and the images were recorded and analyzed using ImageJ. Meanwhile, RAW 264.7 cells treated with a Gel/Zr/EGCG hydrogels extract and lipopolysaccharide (LPS, 1 µg mL^−1^) were used to detect the expression levels of interleukin 6 (IL‐6) and tumor necrosis factor α (TNF‐α); LPS‐treated RAW 264.7 cells were used as the control. The total RNA content was extracted by TRIzol using a standard protocol. A complementary DNA (cDNA) template was synthesized by reverse transcription according to the manufacturer's instructions. RT‐qPCR was performed using a Roche light cycler 480 fluorescence quantitative PCR instrument (Roche). The expression levels of the relative target genes were calculated by 2^−ΔΔCt^.

### Acute Wound Healing

The wound‐healing characteristics of the hydrogels were studied using a mouse model (C57). Briefly, male mice weighing 18–21 g were anesthetized, and a circular full‐thickness skin wound (8 mm in diameter) was cut under sterile conditions.^[^ ^51^ ^]^ The wound‐healing rate was calculated by comparing the area of the healing wound with that of the original wound in the different hydrogel groups. After 14 days of healing, the entire wound area and adjacent normal skin were collected and fixed in 4% (polyoxymethylene, POM). After sectioning the paraffin‐embedded tissue, the sections were stained with hematoxylin and eosin (H&E) and Masson's trichrome stain for histological analysis.

### Chronic Wound Healing

The acute wound‐healing properties of the hydrogels were studied in a diabetic mouse model (C57). Male mice weighing 18–21 g was selected. Under aseptic conditions, streptozotocin (STZ, 50 mg kg^−1^) was intraperitoneally injected continuously for 5 days. After 7 days of normal feeding, the blood glucose was measured; a blood glucose level >13 mmol L^−1^ indicated the onset of diabetes.^[^ ^52^ ^]^ The mice were anesthetized and full‐thickness skin wounds 8 mm in diameter were opened. The wound healing rates were calculated by comparing the wound healing areas of the different hydrogel groups with those of the original wound at different times (0, 3, 7, and 14 days). After 14 days of healing, the entire wound surface and adjacent normal skin were collected and fixed with a 4% (polyoxymethylene, POM). Histological analysis was performed following Masson's trichrome and H&E staining. Meanwhile, picrosirius (PS) red, cluster of differentiation 31 (CD31), and cytokeratin 19 (CK19) were analyzed by immunofluorescence staining. Immunohistochemical staining for the interleukin‐ 6 (IL‐6) and tumor necrosis factor‐α (TNF‐α). All sections were scanned under a light microscope. All animal procedures were performed in accordance with the Guidelines for Care and Use of Laboratory Animals of Wenzhou Medical University and approved by the Animal Ethics Committee of Wenzhou Medical University (SYXK 2015‐009).

### In Vivo Biocompatibility Evaluation of Gel /Zr/EGCG1.0

The compatibility of Gel/Zr/EGCG1.0 in C57 mice (8–10 weeks of age, 20–25 g) was evaluated by subcutaneously injecting 100 µL of the hydrogels. Mice injected with PBS were the control group. 7 days after the injection, blood samples were collected for serum biochemical testing. The sera were tested for aspartate aminotransferase (AST), alanine aminotransferase (ALT), blood urea nitrogen (BUN), and creatinine (CRE). The major organs (heart, liver, spleen, lung, and kidney) were stained with H&E and histologically analyzed.

### Statistical Analysis

At least three biological replicates were used for all quantitative measurements. Data are presented as the mean ± SD and one‐way analysis of variance (ANOVA) was used for statistical analyses, with *, **, ***, and **** indicating *p* < 0.05, *p* < 0.01, *p* < 0.001, and *p* < 0.0001, respectively.

## Conflict of Interest

The authors declare no conflict of interest.

## Author Contributions

X.Z., D.Y. contributed equally to this work. Conceptualization: J.S., X.Z., X.Z. Methodology: D.Y., Y.Z., Y.X., X.Z. Investigation: D.Y., Y.Z., H.C., S.Z. Visualization: D.Y., Y.Z., H.C., S.N. Supervision: J.S., L.Z., X.Z. Writing—original draft: X.Z., D.Y., Y.Z. Writing—review & editing: D.Y., Y.X., L.Z., J.S., X.Z.

## Supporting information

Supporting Information

## Data Availability

The data that support the findings of this study are available in the Supporting Information of this article.
