# Supplementary material for: Facile General Injectable Gelatin/Metal/Tea Polyphenol Double Nanonetworks Remodel Wound Microenvironment and Accelerate Healing
Source: Adv Sci (Weinh). 2023 Dec 20;11(9):2305405. doi: 10.1002/advs.202305405 (PMC10916639; doi:10.1002/advs.202305405)
Supplement: Supplementary file 1 — Supporting Information [file ADVS-11-2305405-s001.pdf]

ADVANCED SCIENCE
Open Access

Supporting Information

for *Adv. Sci.*, DOI 10.1002/advs.202305405

Facile General Injectable Gelatin/Metal/Tea Polyphenol Double Nanonetworks Remodel Wound Microenvironment and Accelerate Healing

Xingjie Zan, Dong Yang, Yi Xiao, Yaxin Zhu, Hua Chen, Shulan Ni, Shengwu Zheng, Limeng Zhu, Jianliang Shen* and Xingcai Zhang**

Supplementary Materials

Facile General Injectable Gelatin/Metal/Tea Polyphenol Double Nanonetworks Remodel Wound Microenvironment and Accelerate Healing

Xingjie Zan,[1,2†] Dong Yang,[1,2†] Yi Xiao,[3] Yaxin Zhu,[2] Hua Chen,[4] Shulan Ni,[1,2] Shengwu Zheng,[5] Limeng Zhu,[1,2*] Jianliang Shen[1*], Xingcai Zhang[3*]

[1] National Engineering Research Center of Ophthalmology and Optometry, Eye Hospital, Wenzhou Medical University, Wenzhou, 325027, China.

[2] Wenzhou Institute, Wenzhou Key Laboratory of Perioperative Medicine, University of Chinese Academy of Sciences, Wenzhou, 325001, China.

[3] School of Engineering and Applied Sciences, Harvard University, Cambridge, Massachusetts, 02138, USA.

[4] Department of Thoracic Surgery, The First Affiliated Hospital of Wenzhou Medical University, Wenzhou, 325003, China.

[5] Wenzhou Celecare Medical Instruments Co., Ltd, Wenzhou, 325000, China.

[*] Corresponding authors. Emails: zhulimeng@ucas.ac.cn (Limeng Zhu); shenjl@wiucas.ac.cn (Jianliang Shen); xingcai@seas.harvard.edu (Xingcai Zhang).
[†] These authors contributed equally to this work and are the first co-authors.

This PDF file includes:

Figs. S1 to S13
Table. S1

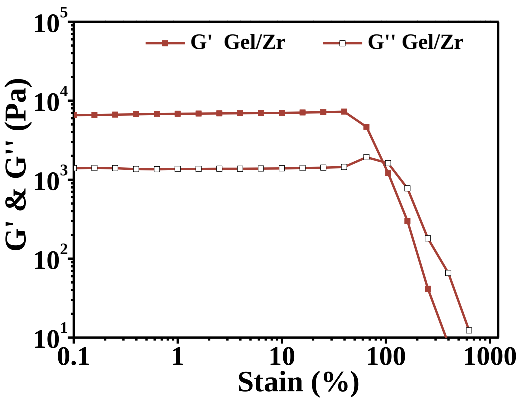

Fig. S1. The Strain amplitude sweep test of Gel/Zr hydrogel.

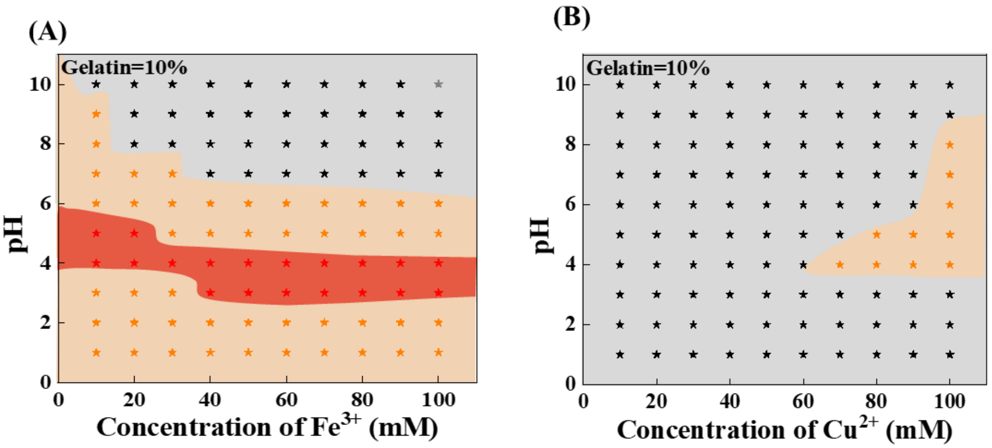

Fig. S2. Phase diagram of metal ions and Gel. (A) Gel and Fe^{3+} mixtures at different pH. **(B)** Gel and Cu^{2+} mixtures at different pH. According to the different colors of stars, they represent liquid (★), coacervate (★), and hydrogel (★).

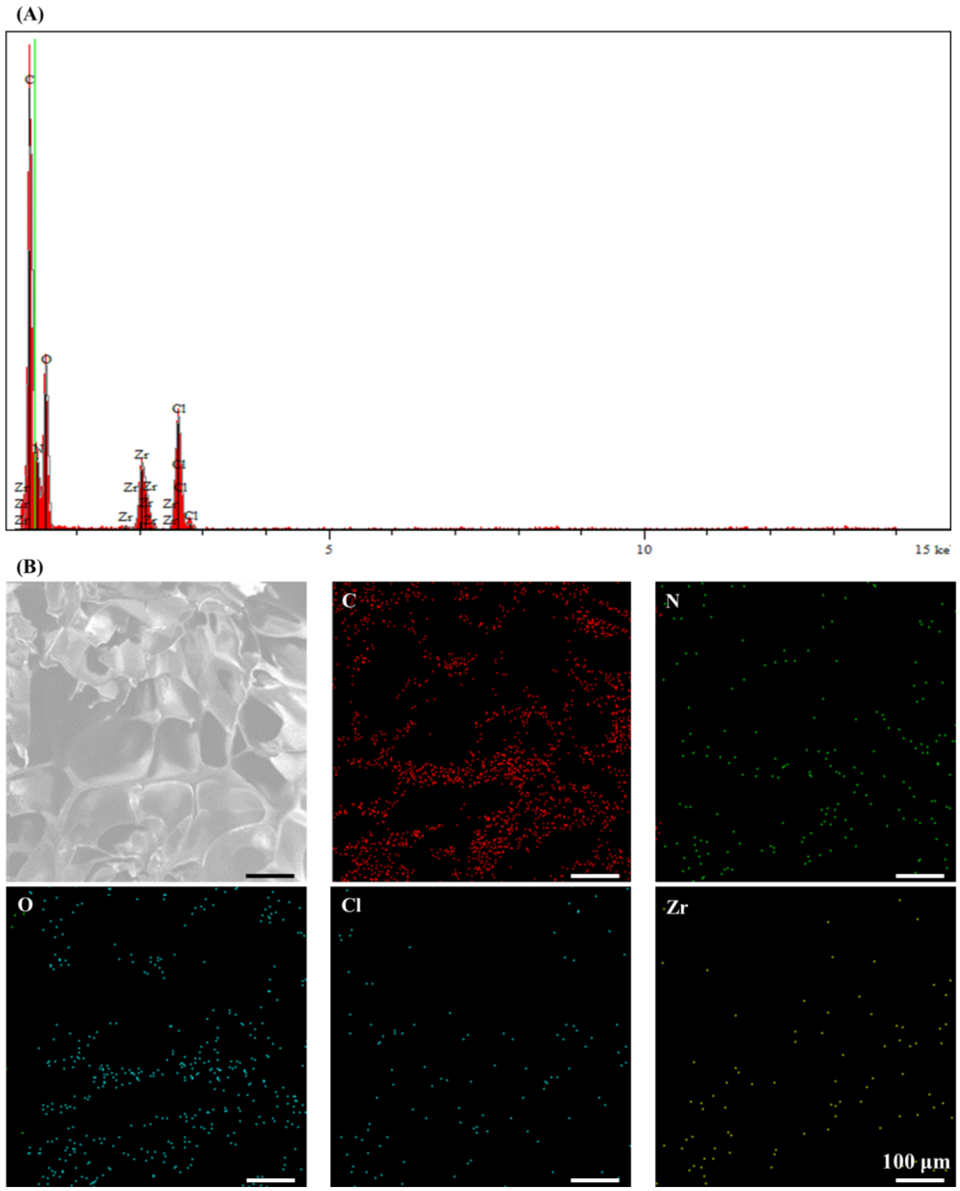

Fig. S3. Elemental analysis by EDS under SEM. (A) The EDS spectra of Gel/Zr hydrogel. **(B)** EDS mapping of Gel/Zr hydrogel.

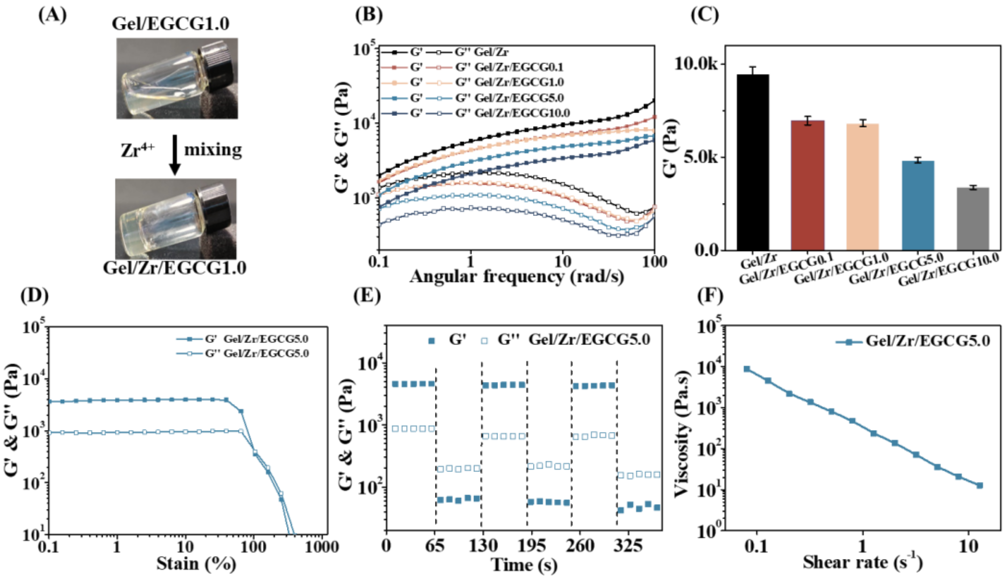

Fig. S4. Fabrication and Viscoelastic characterization of injectable Gel/Zr/EGCG hydrogels. (A) The images of Gel/Zr/EGCG1.0 hydrogel. **(B)** The storage modulus G' and loss modulus G" of Gel/Zr/EGCG hydrogels (strain = 1%). **(C)** The G' values of Gel/Zr/EGCG hydrogels with different EGCG concentrations (The contents of EGCG in the hydrogel are 0.1, 1.0, 5.0, and 10.0 mg/mL for Gel/Zr/EGCG0.1, Gel/Zr/EGCG1.0, Gel/Zr/EGCG5.0, and Gel/Zr/EGCG10.0, respectively). **(D)** The Strain amplitude sweep test of Gel/Zr/EGCG5.0 hydrogel. **(E)** Self-healing properties of Gel/Zr/EGCG5.0 hydrogel under alternating strain from 1% to 400% (frequency = 0.1 Hz). **(F)** Shear-thinning behaviors of Gel/Zr/EGCG5.0 hydrogel.

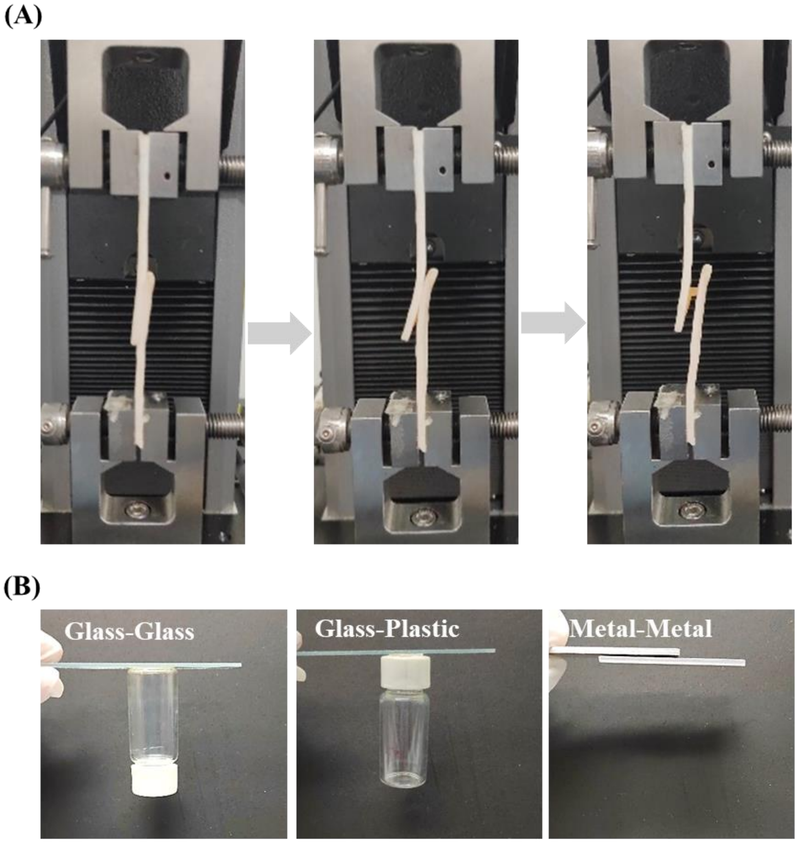

Fig. S5. Photo of Gel/Zr/EGCG1.0 hydrogel adhesion behavior. (A) Schematic lap shear test of Gel/Zr/EGCG1.0 hydrogel on pig skin. **(B)** Pictures of Gel/Zr/EGCG1.0 hydrogel adhesion in different media.

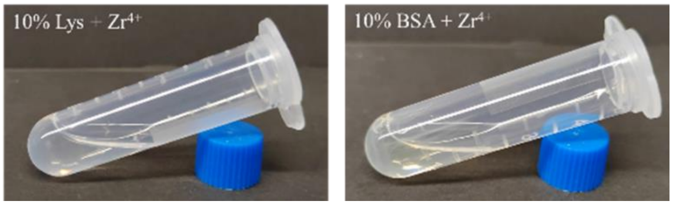

Fig. S6. Photograph of Zr^{4+} mixed with lysozyme (Lys) and bovine serum albumin (BSA).

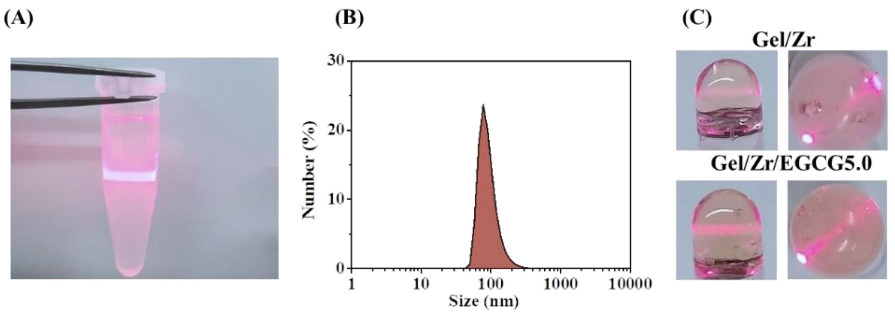

Fig. S7. Characterization of Zr^{4+}/EGCG nanoparticles. (A) Tyndall effect of nanoparticles formed by coordination of Zr^{4+} with EGCG under red laser irradiation. **(B)** Hydrodynamic size distribution of Zr^{4+}/EGCG. **(C)** Red laser was passed through the Gel/Zr and Gel/Zr/EGCG hydrogels, respectively.

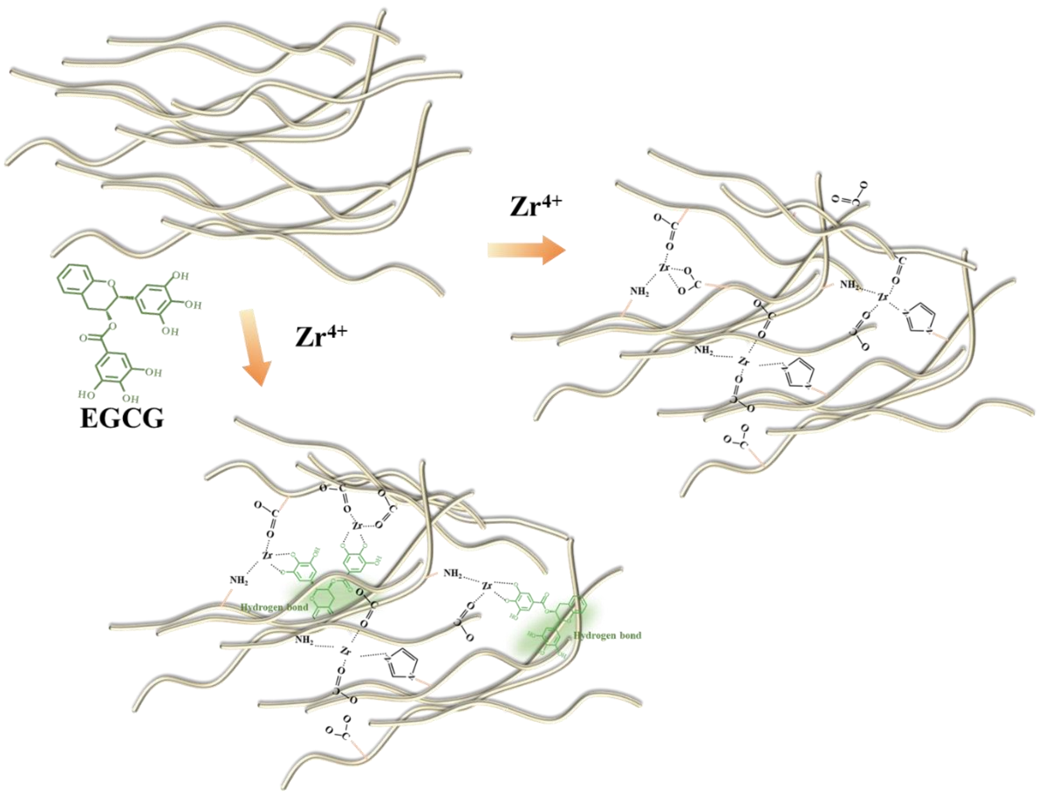

Fig. S8. Schematic of the coordination chemistry of Zr^{4+} with gelatin and EGCG.

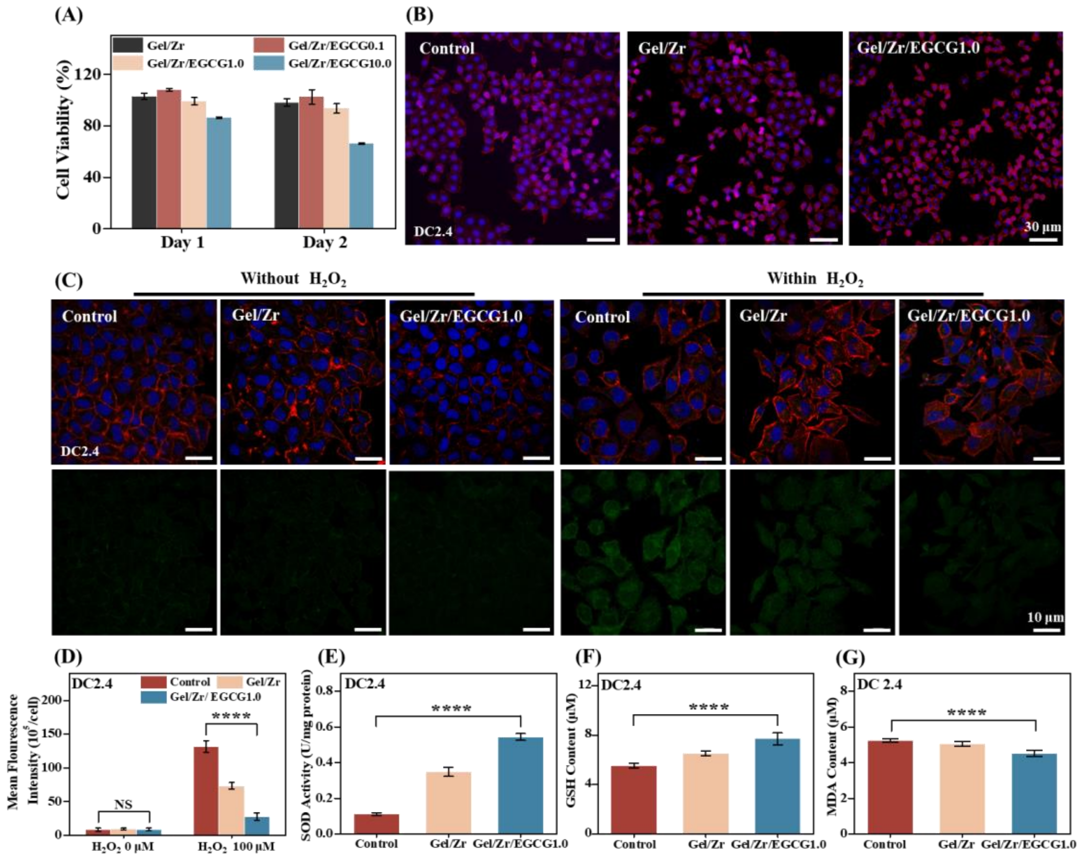

Fig. S9. *In vitro* biocompatibility and intracellular antioxidant capability of Gel/Zr/EGCG1.0 hydrogel. (A) The viability of DC2.4 cells cultured in Gel/Zr and Gel/Zr/EGCG hydrogel extracts after one and two days, respectively (n = 5). **(B)** Fluorescence images of DC2.4 cells co-cultured with Gel/Zr/EGCG1.0 hydrogel for two days (The nucleus is blue and the cytoskeleton is red). **(C)** Intercellular ROS scavenging capability of hydrogels evaluated by DCFH-DA (green fluorescence). **(D)** Statistics of fluorescence intensity for DCFH-DA (n= 4). **(E)** SOD level analysis results by H_2O_2 stimulation (n= 4). **(F)** GSH/GSSG level analysis results by H_2O_2 stimulation (n= 4). **(G)** MDA level analysis results by H_2O_2 stimulation (n= 4).

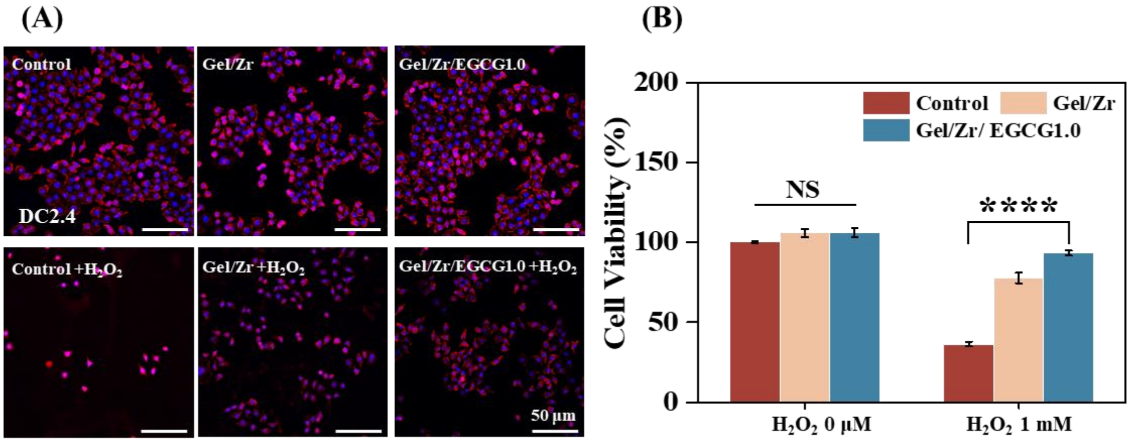

Fig. S10. Viability and morphology of DC2.4 cells. (A) Cytoskeletal structure of DC2.4 cells after incubation in the solution containing hydrogels and H_2O_2. **(B)** DC2.4 Cells survival rate measured by CCK8 kit after incubation in the solution containing hydrogels and H_2O_2 (n = 3).

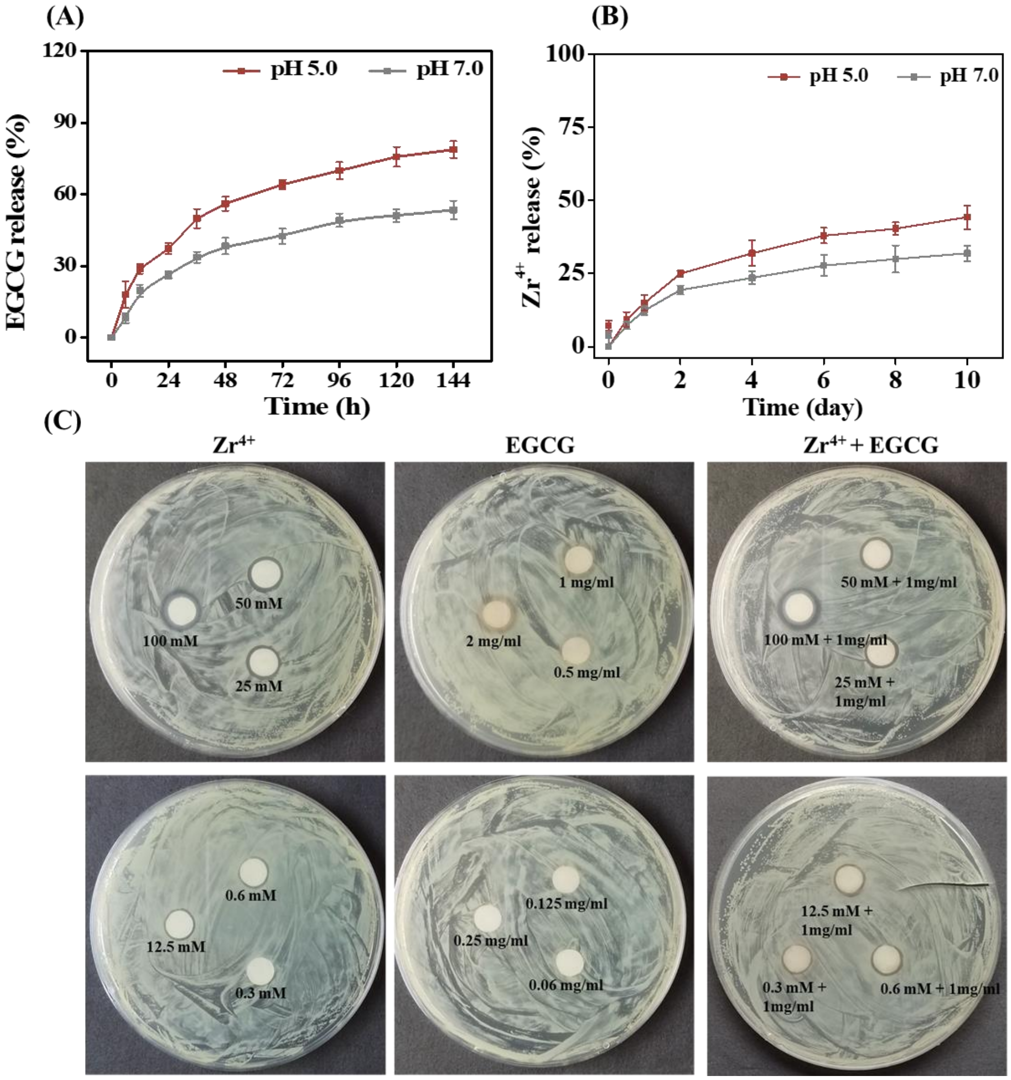

Fig. S11. ***In vitro*** **release experiments of EGCG and Zr from hydrogels, and the circle of inhibition of different concentrations of EGCG, Zr^{4+}, and Zr^{4+}/EGCG.** (A) EGCG release from Gel/Zr/EGCG1.0 hydrogels at different pH conditions (n = 3). (B) Zr^{4+} release from Gel/Zr/EGCG1.0 hydrogels at different pH conditions (n = 3). (C) antibacterial sensitivity with agar diffusion test. A digital camera was used to collect images of the Zr^{4+}, EGCG, Zr^{4+}/EGCG samples different concentrations of Staphylococcus aureus. Summarized data showed the difference in inhibition zone diameters for Staphylococcus aureus among the different groups.

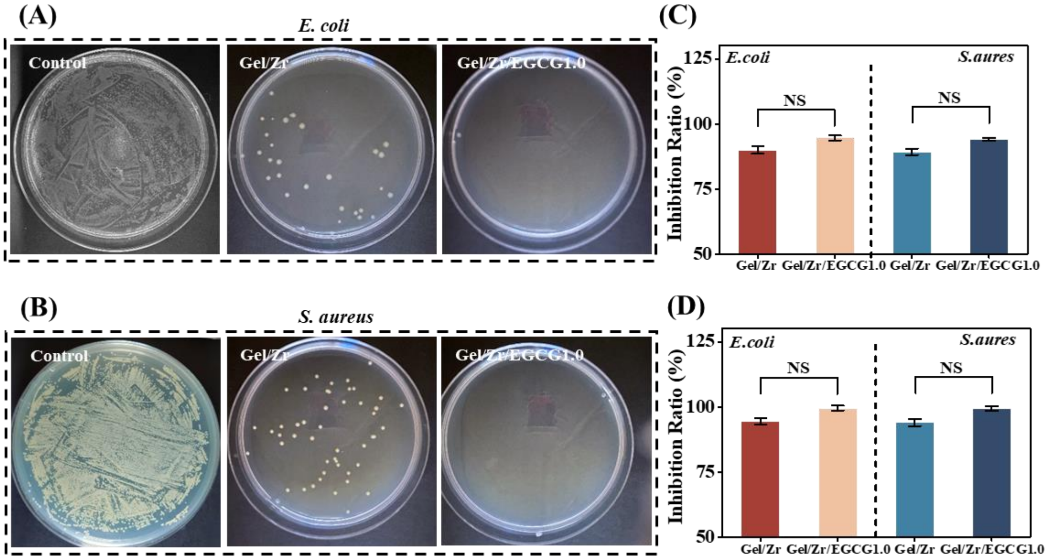

Fig. S12. *In vitro* antibacterial test. (A) *E. coli* survival diagram and **(B)** *S. aureus* survival diagram. **(C)** Histogram of the number of colonies on an agar plate. **(D)** The bacteriostasis rate of the indicated hydrogel at OD $_{600}$ nm was *E. coli and S. aureus* (n = 3).

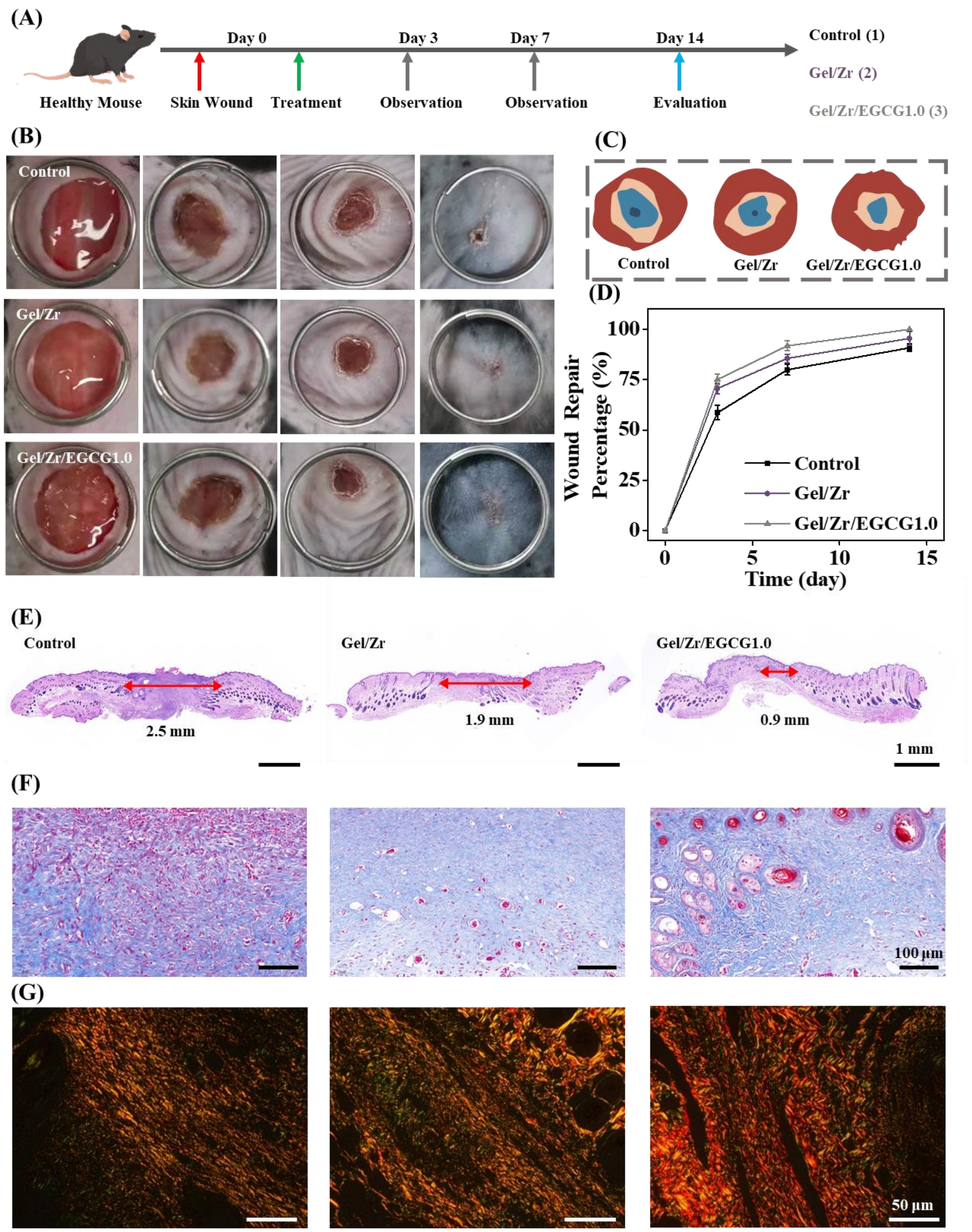

Fig. S13. Wound healing and histopathological images of wound tissue in the healthy mice. **(A)** Schematic illustration for acute wound generation and the subsequent healing process. **(B)** Representative photographs of acute wound healing on days 0, 3, 7, and 14. **(C)** Wound fractions healed by different treatments on days 0, 3, 7, and 14 (n = 3). **(D)** Quantitative analysis of wound area in each group (n = 3). **(E)** H&E staining of the wound area on day 14 (n = 3). **(F)** Partially enlarged Masson images. **(G)** Representative scarlet staining sections PS red (n = 3).

Table S1.

Hydrogels are formed or not after different metal ions are mixed with 5% and 10% Gel after dissolving in an aqueous solution. "No" means hydrogels cannot be formed, and "Yes" means hydrogels can be formed.

		Gelatin concentration (Wt%)	
**Mental ions**	**Concentration（mM）**	**5**	**10**
**Zn^{2+}**	50	No	No
**Ag^{3+}**	50	No	No
**Ce^{3+}**	50	No	No
**Co^{2+}**	50	No	No
**Ca^{2+}**	50	No	No
